# Investigation of circulating lncRNAs as potential biomarkers in chronic respiratory diseases

**DOI:** 10.1186/s12967-020-02581-9

**Published:** 2020-11-10

**Authors:** Zsófia Gál, András Gézsi, Ágnes F. Semsei, Adrienne Nagy, Monika Sultész, Zsuzsanna Csoma, Lilla Tamási, Gabriella Gálffy, Csaba Szalai

**Affiliations:** 1 Department of Genetics, Cell- and Immunobiology, Semmelweis University, Budapest, Hungary; 2 Department of Measurements and Information Systems, Budapest University of Technology and Economics, Budapest, Hungary; 3 MTA-SE Immune-Proteogenomics Extracellular Vesicle Research Group, Semmelweis University, Budapest, Hungary; 4 Heim Pál Children’s Hospital, Budapest, Hungary; 5 National Korányi Institute of TB and Pulmonology, Budapest, Hungary; 6 Department of Pulmonology, Semmelweis University, Budapest, Hungary; 7 Pulmonology Hospital, Törökbálint, Hungary

**Keywords:** Allergic rhinitis, Asthma, COPD, lncRNA, OIP5-AS1, JPX, Biomarker

## Abstract

**Background:**

In the present study the blood expression level of inflammatory response and autoimmunity associated long non-coding RNAs (lncRNAs) were compared in patients with different chronic respiratory diseases and investigated whether they could be used as biomarkers in these diseases.

**Methods:**

In the discovery cohort, the gene expression level of 84 lncRNAs were measured in the blood of 24 adult patients including healthy controls and patients with asthma and COPD. In the replication cohort the expression of 6 selected lncRNAs were measured in 163 subjects including healthy controls and adults with allergic rhinitis, asthma, COPD and children with asthma. It was evaluated whether these lncRNAs can be used as diagnostic biomarkers for any studied disease. With systems biology analysis the biological functions of the selected lncRNAs were predicted.

**Results:**

In the discovery cohort, the mean expression of 27 lncRNAs showed nominally significant differences in at least one comparison. *OIP5-AS1, HNRNPU, RP11-325K4.3, JPX, RP11-282O18.3, MZF1-AS1* were selected for measurement in the replication cohort. Three lncRNAs (*HNRNPU, RP11-325K4.3, JPX*) expressed significantly higher in healthy children than in adult controls. All the mean expression level of the 6 lncRNAs differed significantly between adult allergic rhinitis patients and controls. *RP11-325K4.3, HNRNPU* and *OIP5-AS1* expressed higher in allergic asthma than in non-allergic asthma. COPD and asthma differed in the expression of *RP11-325K4.3* from each other. In examining of the lncRNAs as biomarkers the weighted accuracy (WA) values were especially high in the comparison of healthy controls and patients with allergic rhinitis. *OIP5-AS1* and *JPX* achieved 0.98 and 0.9 WA values, respectively, and the combination of the selected lncRNAs also resulted in a high performance (WA = 0.98). Altogether, *OIP5-AS1* had the highest discriminative power in case of three out of six comparisons.

**Conclusion:**

Differences were detected in the expression of circulating lncRNAs in chronic respiratory diseases. Some of these differences might be utilized as biomarkers and also suggest a possible role of these lncRNAs in the pathomechanism of these diseases. The lncRNAs and the associated pathways are potential therapeutic targets in these diseases, but naturally additional studies are needed for the confirmation of these results.

## Background

Chronic respiratory diseases such as asthma, chronic obstructive pulmonary disease (COPD) and allergic rhinitis cause an enormous burden on the societies and are considered as major non-communicable diseases [1]. Over 1 billion people in the world suffer from chronic respiratory diseases [2, 3]. Patients with these diseases can have a profound impairment in their quality of life and work or school performance. During the last decades, the prevalence of chronic respiratory diseases has dramatically increased. While at the beginning of the twentieth century allergy was considered as a rare disease, today the most common form of allergic disease, allergic rhinitis, has a prevalence of about 25% in Europe, and within the next few decades more than half of the European population will have some type of allergy [4–6]. Asthma, which is a complex chronic inflammatory disease accompanied by episodic airway obstruction and inflammation of the lower respiratory tract affects an estimated 358 million people worldwide and it is the most frequent chronic disease in children [7]. COPD is characterized by airway remodeling which is irreversible in most cases and undergoes progressive changes in contrast to the reversible narrowing of airways in asthma. COPD has an estimated annual death rate of over 4 million people globally [8, 9].

While rhinitis is characterized by an inflammation of the upper airways, asthma and COPD are featured by an inflammation of the lower airways. Although these are separate disease entities, there is a considerable overlap between them [10]. Often allergic rhinitis can develop to asthma and most asthmatic children and a considerable portion of adults have both diseases [11–13]. Patients with COPD can also have asthma, which is called asthma-COPD overlap syndrome, or ACOS [14]. Recent epidemiological data show that COPD is also associated with chronic rhinitis which is a high-risk comorbidity for 30-day hospital re-admission of patients with both asthma and COPD [15–17].

In addition, all these diseases, especially asthma and COPD have several endotypes, i.e. different molecular pathomechanisms can lead to similar phenotypes. Presently, there are no or only very few biomarkers for accurate classification of these diseases or to follow-up the responses to the therapies [9, 15].

Numerous studies have confirmed that 70–90% of the human genome is transcribed into RNA but only 1.2% has protein coding ability. Long non-coding RNAs (lncRNAs) are greater than 200 bp in length, building a major part of non-coding RNAs but in the meantime the least characterized [18]. Depending on the relative position of the sequence of long non-coding gene with respect to the protein-coding region, lncRNAs can be divided into different subgroups including natural antisense (AS), long intergenic (LINC), bidirectional-promoter, enhancer RNAs (eRNAs), promoter associated RNAs (PARs), terminus associated RNAs (TARs) and intronic (INT) lncRNAs [19, 20]. They can participate in cell proliferation, differentiation, processes of programmed cell death and immune response by their capability of binding DNA, RNA and proteins and thereby influencing the transcription process, chromatin remodeling, activity of mRNA and miRNA, localization and structure of proteins [21]. The altered expression of lncRNAs can play a role in various diseases including chronic respiratory diseases. LncRNAs show much greater cell-type specific expression pattern than mRNAs. It was also observed that disease-associated lncRNAs exhibit far greater differences in expression than disease-associated mRNAs and in this way lncRNAs are considered as potential biomarkers [22]. In addition, identifying lncRNAs associating with diseases or disease endotypes can contribute to the understanding of the pathomechanisms of these diseases.

In the present study, first we measured the gene expression level of 84 inflammatory response and autoimmunity associated lncRNAs in the blood of patients with mild or moderate (Global Initiative for Asthma (GINA) 1–3) and severe (GINA 4–5) asthma, COPD and control patients (discovery cohort). Then, based on these results and the scientific literature we selected 6 lncRNAs and compared their expression in an expanded population of patients with different chronic respiratory diseases including pediatric and adult asthma, mild and severe asthma, COPD, allergic rhinitis and in corresponding healthy controls. We also compared the expression of these lncRNAs in different subgroups of these diseases and investigated whether they could be used as biomarkers. Finally, we performed a systems biology analysis aiming to predict the biological functions associated with these lncRNAs.

## Patients and methods

### Study population

Our research consisted of two stages. In the discovery cohort, 24 adult patients were involved, out of which 6 had mild or moderate asthma (GINA 1–3), 6 severe asthma (GINA 4–5), 6 COPD and 6 were healthy controls. Participants with asthma were recruited from Asthma ambulance of National Korányi Institute of TB and Pulmonology and from the Department of Pulmonology of Semmelweis University. Asthmatic subjects were diagnosed based on Global Initiative for Asthma (GINA) guidelines (www.ginasthma.org), as described previously [23]. COPD diagnosis was determined according to the Global Initiative for Obstructive Lung Diseases (https://goldcopd.org) designation. Control subjects were healthy donors. Some characteristics of these subjects are summarized in Table 1.

**Table 1:** Characteristics of the study subjects in the discovery cohort

	Control n = 6	Mild asthma n = 6	Severe asthma n = 6	CODP n = 6
Age (Mean ± SD)	36.7 ± 12	46 ± 19	53.7 ± 19	58.5 ± 12
Gender (Male/Female)	3/3	3/3	0/6	3/3
Eosinophil cell count [%] (Mean ± SD)	–	4.4 ± 5	6.9 ± 6	1.8 ± 3
Neutrophil cell count [%] (Mean ± SD)	–	67 ± 29	61.4 ± 6	74 ± 11
FEV1 [%] (Mean ± SD)	–	88.8 ± 21	72.8 ± 26	59 ± 26
Allergic rhinitis (yes/no)	0/6	3/3	5/1	1/5

The replication cohort consisted of 163 subjects. This cohort included 11 asthmatic children from the Allergology Department of Heim Pál Children’s Hospital, 95 adult patients with asthma, 9 with COPD from the Asthma ambulance of National Korányi Institute of TB and Pulmonology, and from the Department of Pulmonology of Semmelweis University. Out of the asthmatic patients 31 had severe asthma (GINA 4–5) and 64 mild or moderate asthma (GINA 1–3). Adult patients with allergic rhinitis were selected from patients of five Hungarian allergic outpatient centers with documented ragweed allergy with clinical history for at least 2 years with peak symptoms in August–September. Detailed description of this project, named DesensIT can be found elsewhere [24]. These patients had moderate-severe seasonal allergic rhinitis based on Allergic Rhinitis and its Impact on Asthma (ARIA) criteria and their respiratory symptoms remained troublesome despite avoidance or adequate pharmacologic therapy, interfering with usual daily activities or with sleep during the pollen season. The blood was collected outside of the pollen season. The control group consisted of 23 individuals with no history of asthma or allergy. Control children (n = 16) were patients from the Department of Ear, Nose and Throat Medicine of Heim Pál Children’s Hospital. Control adults (n = 7) were healthy donors. More information about the replication cohort can be found in Table 2.

**Table 2:** Characteristics of the study subjects in the replication cohort

	Childhood control	Childhood asthma	Adult control	Adult asthma	COPD	Allergic rhinitis
n (number of samples)	16	11	7	95	9	25
Age (Mean ± SD)	9.6 ± 2	13.5 ± 4	36.4 ± 11	48.3 ± 13	61.6 ± 12	43.5 ± 11
Gender (Male/Female)	8/8	9/2	3/4	39/56	4/5	10/15
Mild or moderate/Severe asthma	–	11/0	–	64/31	–	–
Eosinophil cell count [%] (Mean ± SD)	–	3.7 ± 3	–	5.2 ± 4	3.8 ± 5	–
Neutrophil cell count [%] (Mean ± SD)	–	50.4 ± 25	–	60.7 ± 31	67.2 ± 33	–
FEV1 [%] (Mean ± SD)	–	110.4 ± 16	–	85.8 ± 21	64.3 ± 3	106.4 ± 13
Allergic rhinitis (yes/no)	0/16	10/1	0/7	74/21	3/6	25/0

Subjects were all Caucasian with about 5% Gypsy origin based on Hungarian statistical databases. Written informed consent was provided by all participants or parent/guardian at the time of recruitment. The study was conducted according to the designations determined in the Declaration of Helsinki and approved by the Hungarian Scientific and Research Ethics Committee of the Medical Research Council (ETT TUKEB; Case No.: 3526–0/2010-1018EKU; 14,666–1/2012/EKU; IF-980–9/2016).

### Whole blood collection

Whole blood samples (2.5 ml) were collected in PAXgene Blood RNA Tube (PreAnalitiX, Qiagen, Venlo, The Netherlands) to avoid rapid RNA degradation and to stabilize the intracellular RNA. Thereafter, PAXgene tubes were carefully inverted 8 to 10 times and stored for 2 h to 3 days at room temperature before long-term storage in freezer (− 20 °C).

### Total RNA isolation and reverse transcription

Prior to RNA extraction, after tubes were removed from − 20 °C, they were allowed to thaw and incubated at room temperature for 2 h. RNA purification was carried out according to the protocol in the manual of PAXgene Blood RNA Kit. RNA concentrations were measured using a NanoDrop ND-1000 spectrophotometer (NanoDrop Technologies, Wilmington, DE, USA) and the purity of RNA was determined based on the A_260_/A_280_ ratio, 1.8–2.2 was accepted as pure.

In the discovery cohort, before the reverse transcription, due to the low amount of isolated RNA, amplification was carried out with RT^2^ PreAMP cDNA Sythesis Kit (Qiagen, Hilden, Germany). In the replication study, because of the TaqMan Non-Coding experimental design, High Capacity RNA-to-cDNA Kit (Thermo Fisher, Waltham, MA, USA) was used.

### LncRNA PCR array and assay

The pre-amplified cDNA was measured with a prefabricated Human RT^2^ lncRNA PCR Array (LASH-004Z, Qiagen, Hilden, Germany). This panel consists of 84 verified, pre-validated primer pairs specific for the target genes associated with inflammatory response and autoimmunity. The array also contains 5 primer pairs for housekeeping genes (*SNORA73A, RN7SK, RPLP0, B2M, ACTB*), 1 for the detection of human genomic DNA contamination, 3 for reverse transcription control and 3 for positive PCR control. RT^2^ SYBR Green ROX qPCR Master Mix (Qiagen, Hilden, Germany) was used for real-time PCR reaction on Applied Biosystems 7900HT instrument with 96-Well Block Module.

In the replication cohort, expression level of 6 lncRNAs and 2 reference genes were detected with TaqMan Non-coding RNA Assays (*JPX*: Hs0139517_g1; *AC016629.8*: Hs03678951_m1; *HNRNPU*: Hs00402532; *OIP5-AS1*: Hs01587687_g1; *RP11-282O18.3*: Hs00416786_m1; *RP11-325K4.3*: Hs01594146_s1) and Gene Expression Assays (*B2M*: Hs99999907_m1; *RPLP0*: Hs00420895_gH) with Gene Expression Master Mix (all from Applied Biosystems, Waltham, MA, USA). The measurements were performed in duplicate on 384-well plate (ABI 7900HT) in 10 μl of total PCR Reaction Mix volume.

## Statistical analysis

### Analysis of differential expression

All statistical analyses were performed using R statistical software (R Foundation for Statistical Computing, Vienna, Austria; version 3.6.3). Normalized RNA expression levels were calculated using the formula 2^−ΔCt^, where $\Delta Ct$ = $Ct$(target RNA) − $\stackrel{-}{Ct}$(normalizing factor); $Ct(.)$ is the threshold cycle value of a given gene and $\stackrel{-}{Ct}\left(.\right)$ is the arithmetic mean of the threshold cycle values of certain genes. For the discovery cohort, all five housekeeping genes contained by the prefabricated Human RT^2^ lncRNA PCR Array were used for normalization. For the validation, *B2M* and *RPLP0* were utilized as reference genes because of their relatively stable level of expression. Statistical differential expression of lncRNAs was determined by the Limma package [25]. For that, a linear model was fitted for each lncRNA based on the subgroup of the patients. Then, moderated t-statistics and log-odds of differential expression were calculated by empirical Bayes moderation of the standard errors towards a common value. The resulting nominal p-values were corrected for multiple testing using the Benjamini–Hochberg procedure for each comparison. LncRNAs were considered to be differentially expressed when the adjusted p-value was below 0.05. Principal component analysis of lncRNA expression data was performed with the prcomp function of R.

### Analysis of the lncRNAs as diagnostic biomarkers

To analyze the potential usefulness of the six lncRNAs (chosen for the replication cohort) as diagnostic biomarkers in the studied chronic respiratory diseases, we created Naïve Bayesian classifiers using the e1071 package in R [26]. The models were based on the normalized expression levels of different lncRNA combinations, namely using (1) each lncRNA alone, (2) all six lncRNAs, and (3) only those that showed statistically significant expression differences in case of a particular comparison.

As the number of patients varied highly in the different subgroups, we assessed the performance of the classification models by computing their weighted accuracy (a.k.a. balanced accuracy) defined by the following formula:

$$weighted\;accuracy = \frac{1}{2}\left( {\frac{{TP}}{{TP + FN}} + \frac{{TN}}{{TN + FP}}} \right),$$

where one of the classes (i.e. patient subgroups) is considered “positive”, and the other “negative”, and TP is the number of true positives, TN is the number of true negatives, FN is the number of false negatives, and FP is the number of false positives in the confusion matrix. This formulation assesses the accuracy for each class and weighs them equally independently from the number of samples belonging to the class.

We calculated the confusion matrix for each model by a leave-one-out cross-validation scheme as the following: For a given comparison, we left out one sample and trained the model using all other samples. Next, we predicted the class of the left-out sample using a default cut-off of probability 0.5, and compared the predicted class label with the true one of that sample. Each step of this procedure yielded one element of the confusion matrix based on which we computed the weighted accuracy as described above.

### Prediction of the putative functions of lncRNAs

We performed a systems biology analysis to identify the putative functional pathways and Gene Ontology terms associated with each of the six lncRNAs (chosen for the replication cohort). The overview of this analysis can be seen in Additional file 1 and the detailed description of the process in the Additional file 14.

## Results

### Results of the discovery study

In the discovery group, using a prefabricated human inflammatory response and autoimmunity array, the expression levels of 84 lncRNAs were measured in the blood of 6 patients with mild or moderate asthma (GINA 1–3), 6 with severe asthma (GINA 4–5), 6 with COPD, and in 6 healthy controls (Table 1). Based on the quality controls, the results of a COPD patient were excluded from the evaluation. The heatmap of the relative expression of the lncRNAs (ΔCt values relative to the reference lncRNA genes) in each sample is depicted in Additional file 2. No lncRNA showed statistically significant differential expression between the two genders in any disease group (data not shown). Among the lncRNAs on the panel, there were 2 lncRNAs which showed inherently different expressions in the two genders: *XIST*, which is involved in the inactivation of the X chromosome in women, and *NAV2-AS5*, which is mainly expressed in the testis. These two genes were excluded from the selection. Interestingly, there was no such gender dependent difference in the expression of *JPX*, although according to the scientific literature this lncRNA is transcribed within the X-inactivation center and activates the expression of the *XIST* gene [27].

**Fig. 1:**
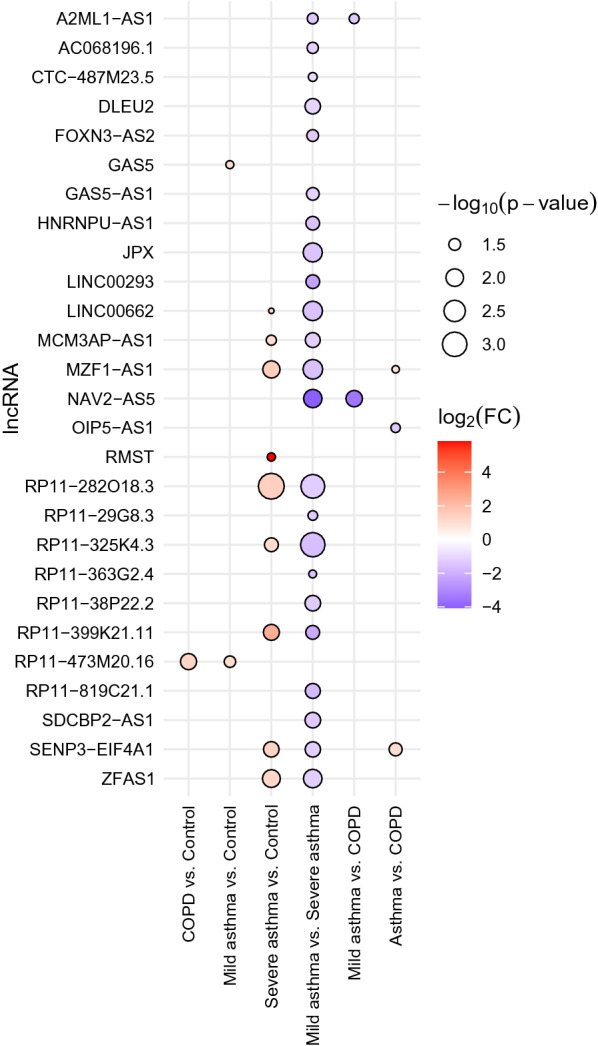
Statistical significance and expression changes of lncRNAs (rows) which showed at least one nominally significant expression difference (P < 0.05) in a given comparison (columns) in the discovery cohort

The expression level of these lncRNAs were compared between different groups of patients. In these comparisons the allergic status of the patients was also considered. According to the phenotypes of the patients, 13 comparisons were made. The compared groups and the heatmap based on the log_2_FC and sex-adjusted P-values can be seen in Additional file 3, Additional file 4 and in Additional file 5. Altogether the mean expression of 27 lncRNAs showed nominally significant differences (P < 0.05) in at least one comparison (Fig. 1). Most differences were found between mild and severe asthma groups. In this comparison 22 out of 84 lncRNAs showed nominally significant differences. Nine lncRNAs showed expression differences between COPD and severe asthma, 3 between asthma and COPD, 3 between asthma and control, 9 between severe asthma and control, 1 between COPD and control, 2 between mild asthma and control groups.

In previous studies two lncRNAs (*OIP5-AS1, HNRNPU*) have been indirectly associated with eosinophil asthma [28, 29]. In our measurements, *HNRNPU* showed increased expression in severe asthma compared with mild asthma, while *OIP5-AS1* showed increased expression in COPD compared to asthma (Fig. 1; Additional file 5). Based on these differences, and data from the scientific literature and databases, 6 lncRNAs were selected for the replication cohort (*OIP5-AS1, HNRNPU, RP11-325K4.3, JPX, RP11-282O18.3, AC016629.8* later renamed to *MZF1-AS1*). It must be added that during our study the transcript variations of the *HNRNPU* which are not translated to a protein and were considered as lncRNAs were withdrawn from the database, in this way, we probably investigated the expression of a protein-coding gene. But, for the sake of simplicity, in this paper, we refer to this gene also as an lncRNA.

### Results in the replication cohort

In the replication cohort 163 patients were involved in 6 different groups (Table 2). In our comparisons the allergic status of the asthmatic patients was also considered and these patients were also stratified according to the severity (GINA 1–3 vs. GINA 4–5), in this way, 10 different groups were created. The results of the comparisons can be seen in Additional file 6 and the heatmap based on the − log_10_P-values of the expression differences between the groups in Additional file 7. The significant differences can be seen in Table 3.

**Table 3:** Log_2_FC and adjusted P-values in the comparison of different groups in the replication cohort in respect of mean blood expression level of lncRNAs. Only those results are given where the adjusted P < 0.05

Comparison	lncRNA	Log_2_FC	Adjusted P value
Adult allergic rhinitis vs. Adult COPD	*RP11-325K4.3*	1.16	0.0002
	*OIP5-AS1*	0.83	0.0013
	*JPX*	0.93	0.0013
	*HNRNPU*	0.72	0.01
	*MZF1-AS1*	0.91	0.01
Adult allergic asthma vs. Adult non-allergic asthma	*RP11-325K4.3*	0.71	0.0007
	*HNRNPU*	0.67	0.0007
	*OIP5-AS1*	0.37	0.0374
Adult allergy vs. Adult allergic asthma	*OIP5-AS1*	0.38	0.0423
Adult asthma vs. Adult COPD	*RP11-325K4.3*	0.82	0.0092
Childhood control vs. Adult control	*HNRNPU*	1.01	0.0079
	*RP11-325K4.3*	0.96	0.0092
	*JPX*	0.85	0.0162
Adult allergic rhinitis vs. Adult asthma	*OIP5-AS1*	0.56	0.0005
	*HNRNPU*	0.43	0.0211
	*JPX*	0.41	0.0226
Adult allergic rhinitis vs. Adult non-allergic asthma	*OIP5-AS1*	0.74	0.0003
	*HNRNPU*	0.76	0.0007
	*RP11-325K4.3*	0.69	0.0025
	*RP11-282O18.3*	0.54	0.0365
	*JPX*	0.44	0.0365
Adult allergic rhinitis vs. Adult control	*OIP5-AS1*	1.11	0.0002
	*JPX*	1.17	0.0003
	*RP11-325K4.3*	1.18	0.0003
	*HNRNPU*	1.10	0.0004
	*RP11-282O18.3*	1.11	0.0058
	*MZF1-AS1*	0.74	0.0360

All the results can be seen in Additional file 6

Interestingly, three lncRNAs (*HNRNPU, RP11-325K4.3, JPX*) expressed significantly higher in pediatric controls than in adult controls (Additional file 8). Because of this, the results when the two age groups were merged (e.g. in case of asthma) were excluded from the evaluations.

**Fig. 2:**
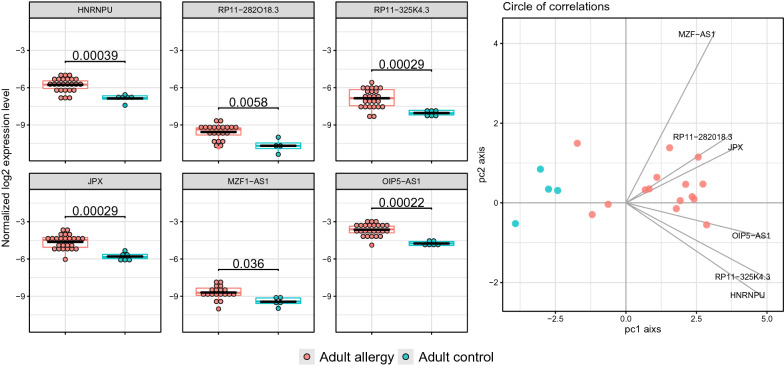
Left: Comparison of the blood expression of the 6 selected lncRNAs between adult patients with allergic rhinitis and controls. The adjusted P-values are given for each comparison. Right: Principal component analysis bi-plot showing the scores of the samples (colored circles) and the loadings of the variables (i.e. the six selected lncRNA as grey arrows) along the first two principal components

**Fig. 3:**
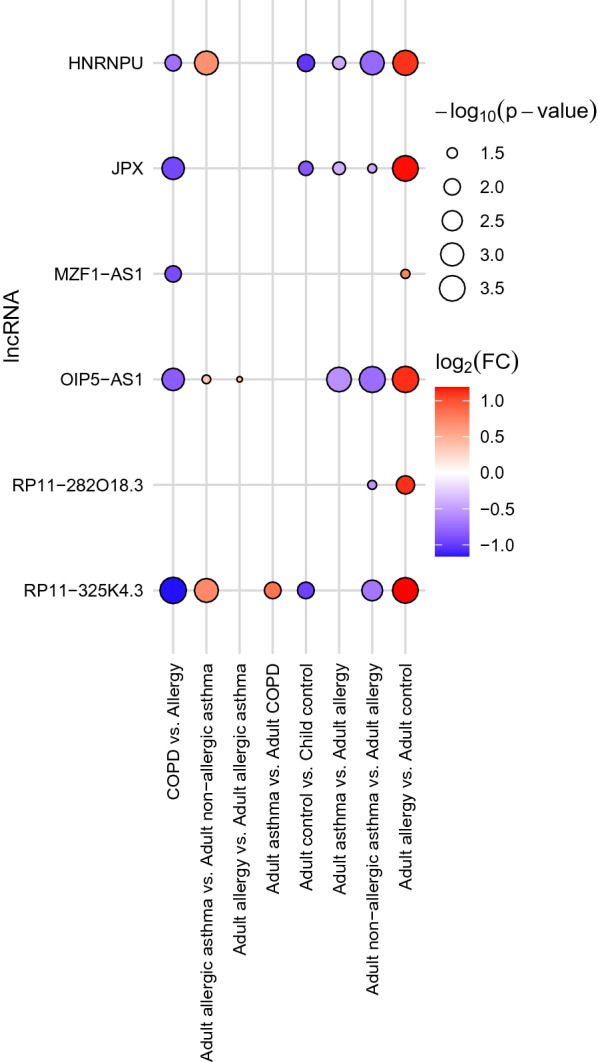
Statistical significance and expression changes of lncRNAs (rows) which showed at least one statistically significant blood expression difference (adjusted P < 0.05) in a given comparison (columns) in the replication cohort

**Fig. 4:**
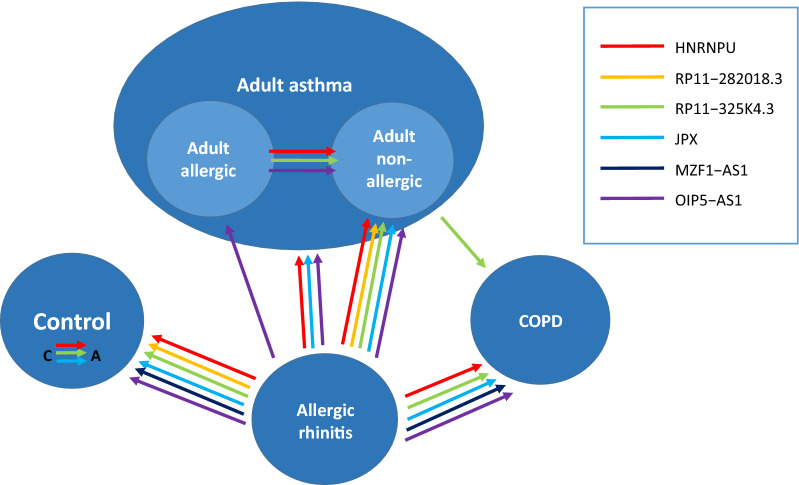
Summary of the different comparisons where at least one of the 6 selected lncRNAs showed statistically significant difference. All arrows indicate significant expression differences. The arrows with different colors denote the lncRNAs, depicted in the right side of the figure, the directions of the arrow indicate the expression levels. The arrows always point to the smaller mean blood expression levels. In the control group C stands for child, A for adult

The most and largest differences were found between adult allergic rhinitis and control patients (Fig. 2). In these cases, the mean expression levels of all 6 lncRNAs differed significantly between the two groups. Principal component analysis indicated that the lncRNAs could be separated into two distinct, uncorrelated groups, namely *OIP5-AS1, HNRNPU, RP11-325K4.3* and *JPX, RP11-282O18.3, MZF1-AS1*, in which the lncRNAs correlated with each other (c.f. the orthogonal loading vectors of lncRNAs in the right panel of Fig. 2). However, in respect of allergy, *OIP5-AS1* seemed to be the most important, since its mean expression level was significantly higher in all cases, where allergy was involved. It was also higher in allergic patients without asthma than in allergic asthmatic patients. A summary of all results can be seen in Figs. 3, 4. It can be seen in the figures that in respect of these lncRNAs, allergic rhinitis differed most significantly from any other phenotypes. In allergic rhinitis the mean expressions of five lncRNAs (*RP11-325K4.3, OIP5-AS1, JPX, HNRNPU, MZF1-AS1*) were significantly higher than in COPD, three (*OIP5-AS1, HNRNPU, JPX*) than in asthma, five (*OIP5-AS1, HNRNPU, RP11-325K4.3, RP11-282O18.3, JPX*) than in non-allergic asthma and one (*OIP5-AS1*) than in allergic asthma. Adult allergic and non-allergic asthma differed in the expression of three lncRNAs from each other, *RP11-325K4.3, HNRNPU* and *OIP5-AS1* expressed higher in allergic asthma. COPD and asthma differed in the expression of one lncRNA from each other. *RP11-325K4.3* expressed significantly higher in the blood of asthmatics than in patients with COPD. The comparisons where significant differences (adjusted P < 0.05) were found can be seen in Additional file 9–14.

In contrast to the discovery cohort, in this expanded population none of the lncRNAs showed association with asthma severity. No differences were found between pediatric asthma and controls. In the replication cohort similarly to the discovery cohort, *JPX* did not show a gender specific expression.

Next, we also analyzed whether the expression of these lncRNAs differ in different subgroups of asthma. No differences were found when asthmatic patients were stratified according to their lung functions (FEV1 < 80% vs. FEV1 > 80%), inhaled corticosteroid usage (regular vs. non-regular), severity, and controllability (controlled vs. non-controlled). We also tested whether the expression levels of these lncRNAs correlated with the blood eosinophil or neutrophil levels but found no correlation (data not shown).

### LncRNAs as biomarkers

**Fig. 5:**
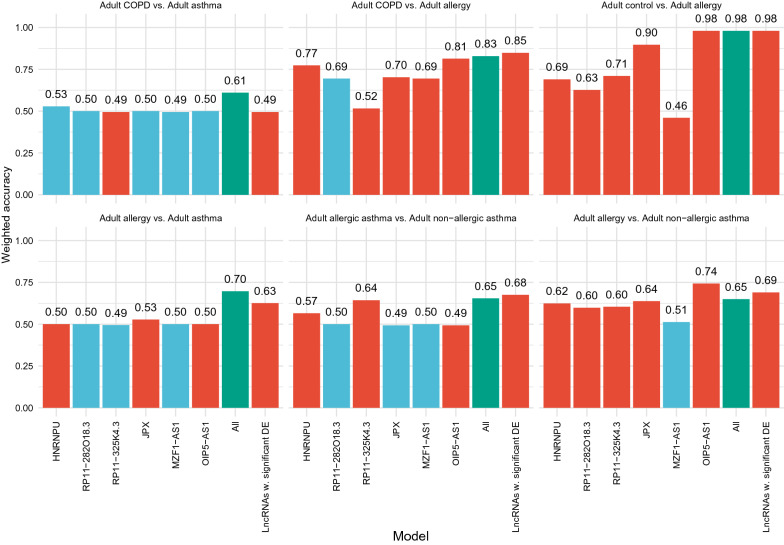
Weighted accuracy of the Naïve Bayesian classification models in different comparisons. Six comparisons were investigated, whether the 6 lncRNAs, alone or in combination were suitable for biomarkers. Naïve Bayesian classifiers based on the normalized expression levels of different lncRNA combinations were created, namely (1) using each lncRNA alone, (2) all six lncRNAs (i.e. the full model), and (3) only those that showed statistically significant expression differences in case of the given comparison (i.e. the reduced model). The performances of the classification models were assessed by computing their weighted accuracy utilizing a leave-one-out cross-validation scheme. Color codes: blue: lncRNAs showing no expression differences between the two groups; red: lncRNAs showing significant differences between the two groups; green: all lncRNAs

Next, we investigated, whether these lncRNAs can be used as diagnostic biomarkers for any studied chronic respiratory disease. The results can be seen in Fig. 5.

Classifying adult allergic rhinitis patients and adult controls, three models achieved a very high performance (WA = 0.98 in case of (1) using *OIP5-AS1* alone, (2) using all six lncRNAs, which is the same model as (3) using all significant lncRNAs with respect to the given comparison). Clearly, these models utilized the high discriminative power of *OIP5-AS1*. Comparing adult COPD and adult patients with allergic rhinitis, using all five significant lncRNAs also resulted in a high performance (WA = 0.85).

In certain cases, combining all six lncRNAs resulted in significantly higher performance than any individual lncRNAs. Comparing adult allergic rhinitis and asthmatic patients, the best model using individual lncRNAs resulted in a WA of 0.53, however, combining all six lncRNAs resulted in a WA of 0.7. Similarly, comparing adult COPD and adult asthmatic patients, the best individual model had a WA of 0.53, and the full model had 0.61, respectively.

In other cases, using the combination of those lncRNAs that showed statistically significant expression differences resulted in a slightly higher performance than the full model. Namely, in case of the aforementioned comparison of adult COPD and adult allergic rhinitis patients, and in case of comparing adult allergic asthmatic and non-allergic asthmatic patients (WA = 0.65 and 0.68 in case of the full model and the reduced model, respectively).

The *OIP5-AS1* lncRNA had the highest discriminative power in case of three out of the six comparisons. Moreover, comparing adult patients with allergic and adult non-allergic asthmatic patients, the model using the individual *OIP5-AS1* had the highest performance of all models (WA = 0.74, which is 5 percent point higher than the second-best model).

### Predicted function of the studied lncRNAs

Finally, we aimed to predict the biological functions associated with the six lncRNAs that were selected for the replication study in order to gain insight into their underlying biological processes (see details in the Additional file 15).

**Fig. 6:**
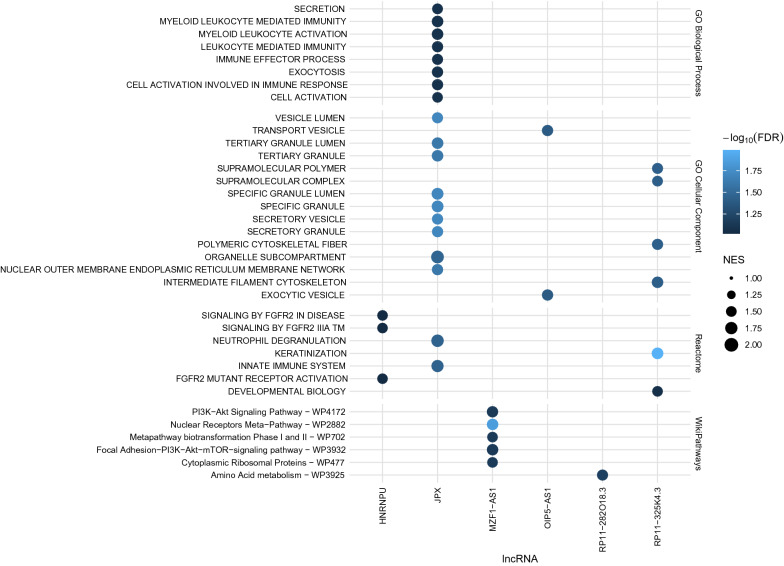
Results of a systems biology analysis of the selected lncRNAs to identify the associated functional biological mechanisms. A network propagation algorithm was initiated from each of the six lncRNAs in a combined heterogeneous lncRNA-gene network to quantitatively prioritize the genes that are expected to be regulated by the given lncRNA. Then, based on the prioritized target genes, gene set enrichment analysis (GSEA) was performed to identify the functionally relevant pathways and Gene Ontology terms. For further details of the method, see the Additional file 15. The color of the dots indicates the statistical significance of the enrichment and the size of the dots indicates the normalized enrichment score for the enriched term in case of the predicted targets of a given lncRNA. FDR: false discovery rate, NES: normalized enrichment score

The results can be seen in Fig. 6. We found no overlap between the statistically significant (FDR < 0.1) predicted functions of the six lncRNAs. *JPX* is predicted to influence several immune-related processes, such as immune effector process (FDR = 0.084), cell activation involved in immune response (FDR = 0.084), the neutrophil degranulation pathway (FDR = 0.035) and the innate immune system pathway (FDR = 0.035). *HNRNPU* is predicted to have an effect on several *FGFR2* related pathways, namely the signaling by FGFR2 in disease pathway (FDR = 0.094), the signaling by *FGFR2* IIIA TM pathway (FDR = 0.094) and the *FGFR2* mutant receptor activation pathway (FDR = 0.094). *MZF1-AS1* is predicted to affect several pathways that regulate cell cycle, cell differentiation/development, proliferation and metabolism, e.g. the PI3K−Akt signaling pathway (FDR = 0.071), the focal adhesion −PI3K−Akt−mTOR−signaling pathway (FDR = 0.071) and the nuclear receptors meta —pathway (FDR = 0.014). *RP11-325K4.3* is predicted to affect developmental processes, such as keratinization (FDR = 0.01). *RP11-282O18.3* is predicted to influence amino acid metabolism (FDR = 0.063). In case of *OIP5-AS1*, the method did not identify any biological processes or pathways. However, it was predicted that genes that are annotated with the transport vesicle and the exocytic vesicle cellular components were significantly enriched among its predicted targets (FDR = 0.04).

## Discussion

In the present study we measured the expression of inflammatory response and autoimmunity associated lncRNAs in the blood of patients with different chronic respiratory diseases. We detected several differences and identified an lncRNA, *OIP5-AS1*, with a very high potency to discriminate patients with severe pollen allergy from non-allergic patients.

In the stage I or discovery study, a smaller number of patients with chronic respiratory diseases and controls were screened with 84 lncRNAs. According to the results of the measurements and data from the scientific literature, 6 lncRNAs were selected for testing on an expanded population. During our study, several studies have been published where the expression of lncRNAs were tested in different chronic respiratory diseases, mainly in asthma, and several differences were found. Some of them were also measured in our stage I study. In our discovery cohort we did not find differences in any comparison in the expression of *TUG1, MALAT1, NEAT1* and *MEG3*, all of them were found to be associated with asthma in different studies [30–33]. Although we measured the expression of these lncRNAs in the blood of only a small number of subjects (6 with mild-moderate, 6 with severe asthma and 6 controls), the lack of differences suggests that they are possibly not suitable for general asthma blood biomarkers. Naturally, they still might play a role in the pathomechanism of asthma in a tissue-specific manner or might be biomarkers for some endotypes or treatment responses. Small, but moderately significant differences were found in the expression of *GAS5* and its antisense *GAS5-AS1* in certain comparisons. These lncRNAs were not selected for the replication study, because the differences were not exceedingly significant (unadjusted P-values were just below the significance level) and at the time of the selection no data about their roles were available in the scientific literature. But, the fact that in a later study the expression of *GAS5* was found to be higher in asthmatics, and knock-down of *GAS5* significantly decreased airway hyperresponsiveness in asthmatic rats, together with our results indicate their possible roles in asthma [34].

The expression of the selected lncRNAs were measured in an expanded population. The largest differences were found between controls and patients with allergic rhinitis. The expression of all selected lncRNAs were significantly higher in patients with allergic rhinitis. Among these, *OIP5-AS1, HNRNPU* and *JPX* are the best studied. Using a combination of three biological networks we also carried out a bioinformatic analysis to predict the biological function and the associated GO terms of these six lncRNAs.

*OIP5-AS1* is a conserved gene acting as a sponge for multiple cellular RNAs and microRNAs, regulating mitosis, maintaining cell proliferation, and functioning as an oncogene in several cancers [35–38]. Interestingly, *OIP5-AS1* by binding to miR-200b, also regulates indirectly the expression of *ACE2*, the receptor for COVID-19, but its implication in the infection has not yet been studied [39]. It was also found to be co-expressed with genes associated with eosinophilic asthma [28, 29], but its role in allergic rhinitis was not yet investigated. Our bioinformatic analysis showed that genes that were annotated with the transport vesicle and the exocytic vesicle cellular components were significantly enriched among the predicted targets of *OIP5-AS1*. In our study its mean expression level was significantly higher in all diseases, where allergy was involved, e.g. in allergic rhinitis vs. COPD or in allergic asthma vs. non-allergic asthma, but its highest level was measured in allergic rhinitis.

The situation with the *HNRNPU* gene is more complicated. It has several aliases in the databases, and earlier it was determined that there are several transcripts from its genome locus, including those that are not translated into a protein (*HNRNPU-AS1,* which were considered as lncRNAs and were on the pre-made array used in our measurement), but recently these have been withdrawn from the databases [40]. In this way the investigated *HNRNPU* gene is probably a protein-coding gene. The function of the protein, however, is similar to several lncRNAs, namely it binds nucleic acids, participates in the formation of ribonucleoprotein complexes in the nucleus with heterogeneous nuclear RNA and plays important role in three-dimensional genome organization. As we have measured gene expression (i.e. RNA), we think that the characteristics of an RNA whether it is translated into a protein or not, cannot influence its possible use as a biomarker, thus we assume that involving this protein-coding gene in the evaluations did not cause bias in our results. HNRNPU was found to be implicated in several processes, including regulation of the innate immunity, proliferation and several diseases like cancers and eosinophilic asthma [28, 29, 41–45]. Our bioinformatic analysis showed that *HNRNPU* was associated with several *FGFR2* related pathways.

The best-known role of *JPX* is that it serves as a molecular switch in the X chromosome inactivation in females, but studies also show that it is implicated in different cancers and can act as an oncogene in certain cases while as a tumor suppressor in others [27, 46]. *JPX* is predicted to influence several immune-related processes, such as immune effector process, cell activation involved in immune response, the neutrophil degranulation pathway and the innate immune system pathway. The lncRNA *MZF1‐AS1* was identified as a transcriptional regulator of proline synthesis and neuroblastoma progression and was associated with several pathways that regulate cell cycle, cell differentiation/development, proliferation and metabolism [47].

In respect of *RP11-325K4.3* and *RP11-282O18.3* until now no publications have been found. Our bioinformatic analysis predicted that RP11-325K4.3 was associated with developmental processes, while *RP11-282O18.3* with amino acid metabolism.

Some of the studied genes (*HNRNPU, RP11-325K4.3, JPX*) showed significantly higher expression in children than in adults. As *HNRNPU* and *JPX* are both implicated in cell proliferation, their increased blood levels in children suggest that they might have roles in their development. The function of *RP11-325K4.3* has not yet been clarified, but its increased level in children also confirms its possible role in developmental processes found in our bioinformatic analysis. It is also noteworthy, that this was the only lncRNA that showed significant difference between adult asthma and COPD. The expression of the investigated genes, however, did not differ between asthmatic children and controls.

According to a study where ageing-associated changes in the expression of lncRNAs in adult human tissues were investigated (between 20 and 79 years of age) no lncRNA was identified in the blood that showed age-dependent expression [48]. It suggests that after reaching adulthood the expression of lncRNAs do not change any more in the blood, and in this way blood expressed lncRNAs in adulthood might be used as age-independent biomarkers. Naturally, this must be tested in larger and diverse populations.

Perhaps, the most interesting finding of this study is the large significant differences between healthy controls and allergic rhinitis patients in the expression of the selected circulating lncRNAs. Until now no paper has been published about the human blood levels of lncRNAs in allergic rhinitis. We also tested whether these lncRNAs are suitable as biomarkers. Those comparisons were analyzed where at least one significant difference was found. For the evaluations the Naïve Bayesian classifiers were used. The selected lncRNAs were tested individually and in combinations. In some cases, the expression levels of the lncRNAs showed highly significant differences between two groups (e.g. *RP11 − 325K4.3* in COPD vs. asthma (adjusted P = 0.0092) and COPD vs. allergic rhinitis (adjusted P = 0.0002)), still its discriminative power, due to its high variance, was low (weighted accuracy (WA) = 0.49 and 0.52, respectively). In these cases, the given lncRNA is not suitable for being a circulating blood biomarker, but these differences suggest that it might have a role in the pathomechanism of one of these diseases or their endotypes. In some cases, however, the lncRNAs alone or in combinations achieved very high performances. The WA values were especially high in the comparison of healthy adult controls and adult patients with allergic rhinitis. *OIP5-AS1* and *JPX* achieved 0.98 and 0.9 WA values, respectively, and the combination of the selected lncRNAs also resulted in a high performance (WA = 0.98). The WA values were also high in the comparison of COPD and allergic rhinitis (WA = 0.85 using the five significant lncRNAs and 0.81 when using *OIP5-AS1* alone), although 30% of the COPD patients also had allergic rhinitis. The WA value was not very high in comparison of allergic vs. non-allergic asthma (0.68 when lncRNAs with statistically significant expression differences were used) but because there is still no solid biomarker in the differential diagnosis of these two endotypes, an additional biomarker might be worth testing [49].

Although the diagnosis of allergic rhinitis is relatively straightforward (e.g. symptoms, skin prick test, allergen-specific IgE), there is still no objective biomarker in allergen specific immunotherapy (AIT) which is able to track how patients respond to the therapy. Presently, the evaluation of clinical improvement is based on changes in subjective clinical and immunological parameters. Different algorithms have been developed for calculating adjusted symptom and medication scores, but none of them is universally accepted [24]. Naturally, it cannot be definitely stated that *OIP5-AS1, JPX* or the combination of these 6 lncRNAs will be useful biomarkers in AIT, but they are worth testing. In 5 of the 6 cases their expression levels were more than twice those of in the controls. Especially the *OIP5-AS1* is quite promising, whose expression level showed relative small variances in both patients and controls, and its discrimination potential, even alone, was very high. It must be noted, however, that the samples were collected in May and June, while the ragweed peak season in Hungary is between August and October. Presently, it is not yet known what the blood levels of these lncRNAs are when the symptoms are serious, and how they change during AIT. But, their significantly higher expressions indicate that they are possibly involved in the pathomechanism of allergic rhinitis and they are potential novel drug targets. E.g. it is well-known that the majority of symptoms in allergy are caused by exocytosis of pre-formed inflammatory mediators-containing granules from mast and basophil cells elicited by FcεRI upon binding of the allergen to receptor bound allergen-specific IgE. According to our bioinformatic analysis *OIP5-AS1* is associated with transport vesicle and exocytic vesicle cellular components. Its higher level in allergic patients might indicate a connection of *OIP5-AS1* with this process suggesting a potential drug or therapeutic target.

Some limitations of the study must also be mentioned. The estimated number of lncRNAs in the human genome is more than 50,000 [50], although their annotations are far from complete (see the case of *HNRNPU-AS1*). In the present study, only 84 selected lncRNAs were involved. Methods with higher capacity (e.g. RNA-seq) additional lncRNAs with larger potentials might be identified. In some groups, the number of study subjects were low. Moreover, in these diseases a lot of additional endotypes exist that were not tested in the present study. Additional, larger studies with more patients with verified, diverse endotypes are needed to utilize the biomarker potential of these lncRNAs and to get better understanding of their roles in these diseases.

## Conclusion

Differences were detected in the expression of circulating lncRNAs in chronic respiratory diseases. Some of these differences might be utilized as biomarkers and also suggest a possible role of these lncRNAs in the pathomechanism of these diseases. With a systems biology analysis, novel functions of some of the lncRNAs were predicted. The lncRNAs and the associated pathways are potential therapeutic targets in these diseases, but naturally additional studies are needed for the confirmation of these results.

## Supplementary information

Additional file 1: Overview of the systems biology analysis to identify the functional pathways and Gene Ontology terms of the 6 selected lncRNAs. A. Construction of a meta-network consisting of two types of meta-nodes, namely lncRNAs and genes; and four meta-edges, namely (1) the tissue-specific transcriptional similarity of lncRNAs, (2) the tissue-specific transcriptional similarity between lncRNAs and genes, (3) the experimentally validated lncRNA-target gene pairs connecting lncRNAs and genes, and (4) protein-protein interaction of genes. B. The heterogeneous lncRNA-gene network induced by the meta-network. Diamond-shaped nodes represent lncRNAs, and circular nodes represent genes. Edges represent functional connection between the corresponding nodes consistent with the meta-edges. C. A random walk with restart network propagation algorithm is initiated from each of the six lncRNAs to quantitatively prioritize the genes that are expected to be functionally relevant with respect to a particular lncRNA. The color of the nodes represent the amount of propagated information in that node (i.e. steady state probability of the random walker visiting that particular node). D. Schematic representation of gene set enrichment analysis on the propagated gene scores.

Additional file 2: Heatmap of the relative expression of the lncRNAs in each sample of the discovery cohort. Color codes above the heatmap: blue: severe allergic asthma; red: mild allergic asthma; green: COPD; yellow: control; brown: non-allergic mild asthma; black: non-allergic severe asthma.

Additional file 3: Heatmap of the log2FC values in comparison of the blood expression of 84 lncRNAs of the study subjects in the discovery cohort.

Additional file 4: Heatmap of the sex adjusted –log10P values in comparison of the blood expression of 84 lncRNAs of the study subjects in the discovery cohort.

Additional file 5: Log2FC and P-values in the comparison of different groups in the discovery cohort in respect of the mean blood expression levels of altogether 84 lncRNAs.

Additional file 6: Log2FC and adjusted P-values in the comparison of different groups in the replication cohort in respect of the mean blood expression levels of the selected lncRNAs. P-values <0.05 are highlighted.

Additional file 7: Heatmap of the adjusted –log10P values in comparison of the blood expression of the 6 selected lncRNAs of the study subjects in the replication cohort.

Additional file 8: Comparison of the selected lncRNAs. Left: Comparison of the blood expressions of the 6 selected lncRNAs between adult and childhood controls. The adjusted P values are given for each comparison. Right: Principal component analysis bi-plot showing the scores of the samples (colored circles) and the loadings of the variables (i.e. the six selected lncRNA as grey arrows) along the first two principal components.

Additional file 9:  Comparison of the selected lncRNAs. Left: Comparison of the blood expression of the 6 selected lncRNAs between groups. The adjusted P-values are given for each comparison. Only those comparisons are depicted, where at least one significant difference was found. Right: Principal component analysis bi-plot showing the scores of the samples (colored circles) and the loadings of the variables (i.e. the six selected lncRNA as grey arrows) along the first two principal components.

Additional file 10: Comparison of the selected lncRNAs. Left: Comparison of the blood expression of the 6 selected lncRNAs between groups. The adjusted P-values are given for each comparison. Only those comparisons are depicted, where at least one significant difference was found. Right: Principal component analysis bi-plot showing the scores of the samples (colored circles) and the loadings of the variables (i.e. the six selected lncRNA as grey arrows) along the first two principal components.

Additional file 11: Comparison of the selected lncRNAs. Left: Comparison of the blood expression of the 6 selected lncRNAs between groups. The adjusted P-values are given for each comparison. Only those comparisons are depicted, where at least one significant difference was found. Right: Principal component analysis bi-plot showing the scores of the samples (colored circles) and the loadings of the variables (i.e. the six selected lncRNA as grey arrows) along the first two principal components.

Additional file 12: Comparison of the selected lncRNAs. Left: Comparison of the blood expression of the 6 selected lncRNAs between groups. The adjusted P-values are given for each comparison. Only those comparisons are depicted, where at least one significant difference was found. Right: Principal component analysis bi-plot showing the scores of the samples (colored circles) and the loadings of the variables (i.e. the six selected lncRNA as grey arrows) along the first two principal components.

Additional file 13: Comparison of the selected lncRNAs. Left: Comparison of the blood expression of the 6 selected lncRNAs between groups. The adjusted P-values are given for each comparison. Only those comparisons are depicted, where at least one significant difference was found. Right: Principal component analysis bi-plot showing the scores of the samples (colored circles) and the loadings of the variables (i.e. the six selected lncRNA as grey arrows) along the first two principal components.

Additional file 14: Comparison of the selected lncRNAs. Left: Comparison of the blood expression of the 6 selected lncRNAs between groups. The adjusted P-values are given for each comparison. Only those comparisons are depicted, where at least one significant difference was found. Right: Principal component analysis bi-plot showing the scores of the samples (colored circles) and the loadings of the variables (i.e. the six selected lncRNA as grey arrows) along the first two principal components.

Additional file 15. Detailed description of the systems biology analysis used for the prediction of lncRNA functions

## Data Availability

Some of the datasets used and/or analyzed during the current study are available as a Additional files 5, 6: Tables S1, S2, any other datasets could be requested from the corresponding author on reasonable request.

## Abbreviations

ACOS: Asthma-COPD overlap syndrome
ACTB: Actin Beta
AIT: Allergen specific immunotherapy
Akt: Protein kinase B
ARIA: Allergic Rhinitis and its Impact on Asthma
AS: Natural antisense
B2M: Beta-2-Microglobulin
COPD: Chronic obstructive pulmonary disease
DNA: Deoxyribonucleic acid
eRNA: Enhancer RNA
FcεRI: Type I high affinity IgE receptor
FDR: False discovery rate
FEV1: Forced expiratory volume in 1 s
FGFR2: Fibroblast growth factor receptor 2
FN: False negative
FP: False positive
GAS5: Growth arrest specific 5
GAS5-AS1: GAS5 antisense RNA 1
GINA: Global initiative for asthma
GO: Gene ontology
HNRNPU: Heterogeneous Nuclear Ribonucleoprotein U
IgE: Immunoglobulin E
INT: Intronic
LINC: Long intergenic
lncRNA: Long non-coding RNA
Log_2_FC: Log2 fold change
MALAT1: Metastasis associated lung adenocarcinoma transcript 1
MEG3: Maternally expressed 3
mRNA: Messenger RNA
mTOR: Mammalian target of rapamycin
MZF1-AS1: Myeloid Zinc Finger 1—antisense RNA 1
NAV2-AS5: Neuron Navigator 2—antisense RNA 5
NEAT1: Nuclear Enriched Abundant Transcript 1
NES: Normalized enrichment score
OIP5-AS1: OPA-interacting protein 5 antisense transcript 1
PAR: Promoter associated RNA
PCA: Principal component analysis
PCR: Polymerase chain reaction
PI3K: Phosphoinositide 3-kinase
RN7SK: RNA Component Of 7SK Nuclear Ribonucleoprotein
RNA: Ribonucleic acid
RPLP0: Ribosomal protein lateral stalk subunit P0
SNORA73A: Small Nucleolar RNA, H/ACA Box 73A
TAR: Terminus associated RNA
TN: True negative
TP: True positive
TUG1: Taurine Up-Regulated 1
WA: Weighted accuracy
XIST: X-inactive specific transcript
