# Supplementary material for: Investigation of circulating lncRNAs as potential biomarkers in chronic respiratory diseases
Source: J Transl Med. 2020 Nov 10;18:422. doi: 10.1186/s12967-020-02581-9 (PMC7653503; doi:10.1186/s12967-020-02581-9)
Supplement: Supplementary file 1 — Additional file 1: Overview of the systems biology analysis to identify the functional pathways and Gene Ontology terms of the 6 selected lncRNAs. A. Construction of a meta-network consisting of two types of meta-nodes, namely lncRNAs and genes; and four meta-edges, namely (1) the tissue-specific transcriptional similarity of lncRNAs, (2) the tissue-specific transcriptional similarity between lncRNAs and genes, (3) the experimentally validated lncRNA-target gene pairs connecting lncRNAs and genes, and (4) protein-protein interaction of genes. B. The heterogeneous lncRNA-gene network induced by the meta-network. Diamond-shaped nodes represent lncRNAs, and circular nodes represent genes. Edges represent functional connection between the corresponding nodes consistent with the meta-edges. C. A random walk with restart network propagation algorithm is initiated from each of the six lncRNAs to quantitatively prioritize the genes that are expected to be functionally relevant with respect to a particular lncRNA. The color of the nodes represent the amount of propagated information in that node (i.e. steady state probability of the random walker visiting that particular node). D. Schematic representation of gene set enrichment analysis on the propagated gene scores. [file 12967_2020_2581_MOESM1_ESM.pdf]

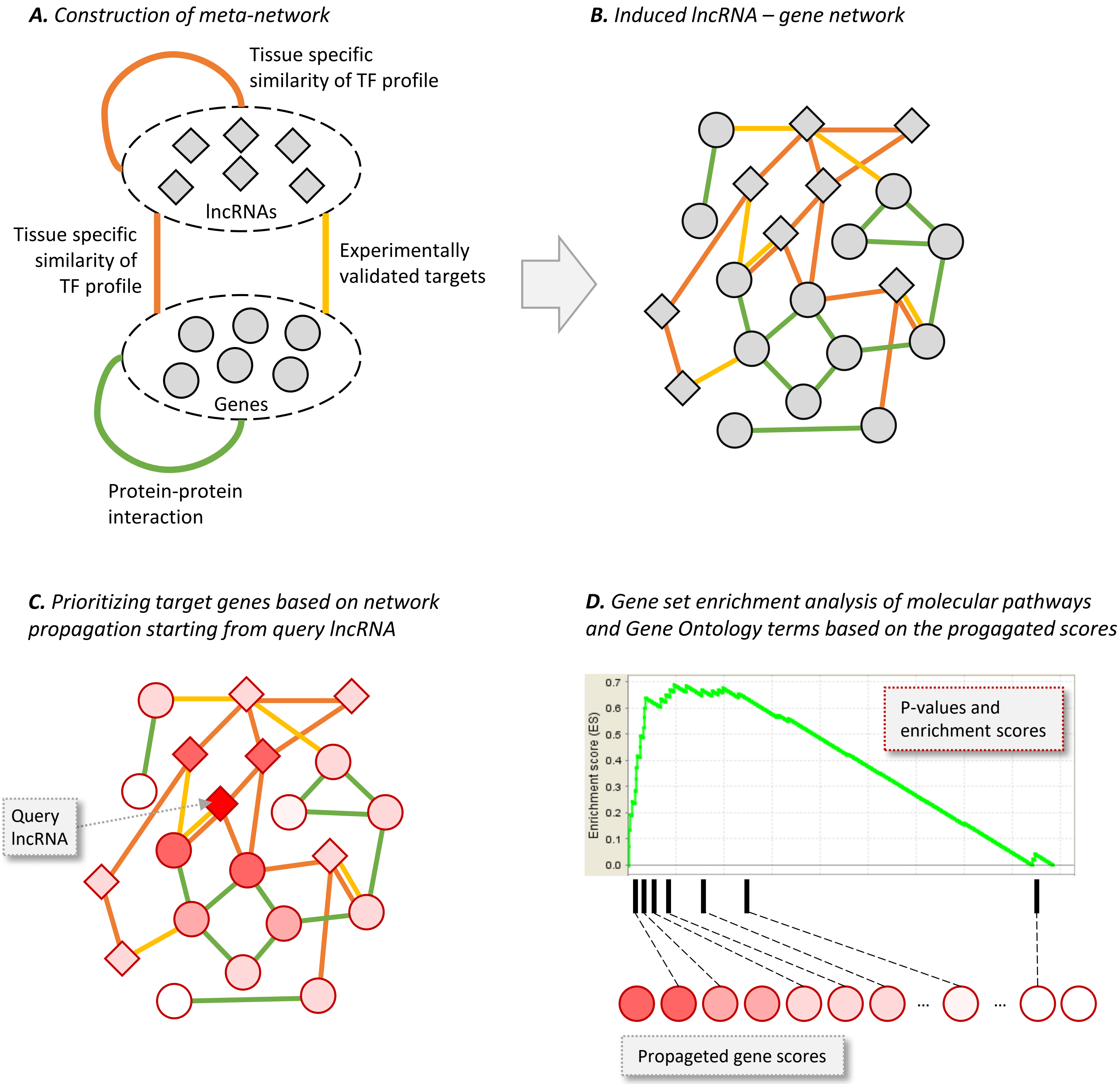
A. Construction of meta-network
Tissue specific similarity of TF profile
lncRNAs
Tissue specific similarity of TF profile
Experimentally validated targets
Genes
Protein-protein interaction
B. Induced lncRNA – gene network
C. Prioritizing target genes based on network propagation starting from query lncRNA
Query lncRNA
D. Gene set enrichment analysis of molecular pathways and Gene Ontology terms based on the progagated scores
Enrichment score (ES)
0.7
0.6
0.5
0.4
0.3
0.2
0.1
0.0
P-values and enrichment scores
...
...
Propageted gene scores
