# Supplementary figures and images for: Investigation of circulating lncRNAs as potential biomarkers in chronic respiratory diseases
Source: J Transl Med. 2020 Nov 10;18:422. doi: 10.1186/s12967-020-02581-9 (PMC7653503; doi:10.1186/s12967-020-02581-9)

Color Key  
and Histogram

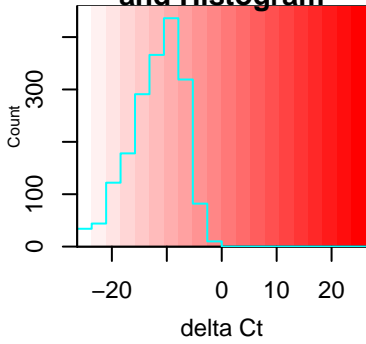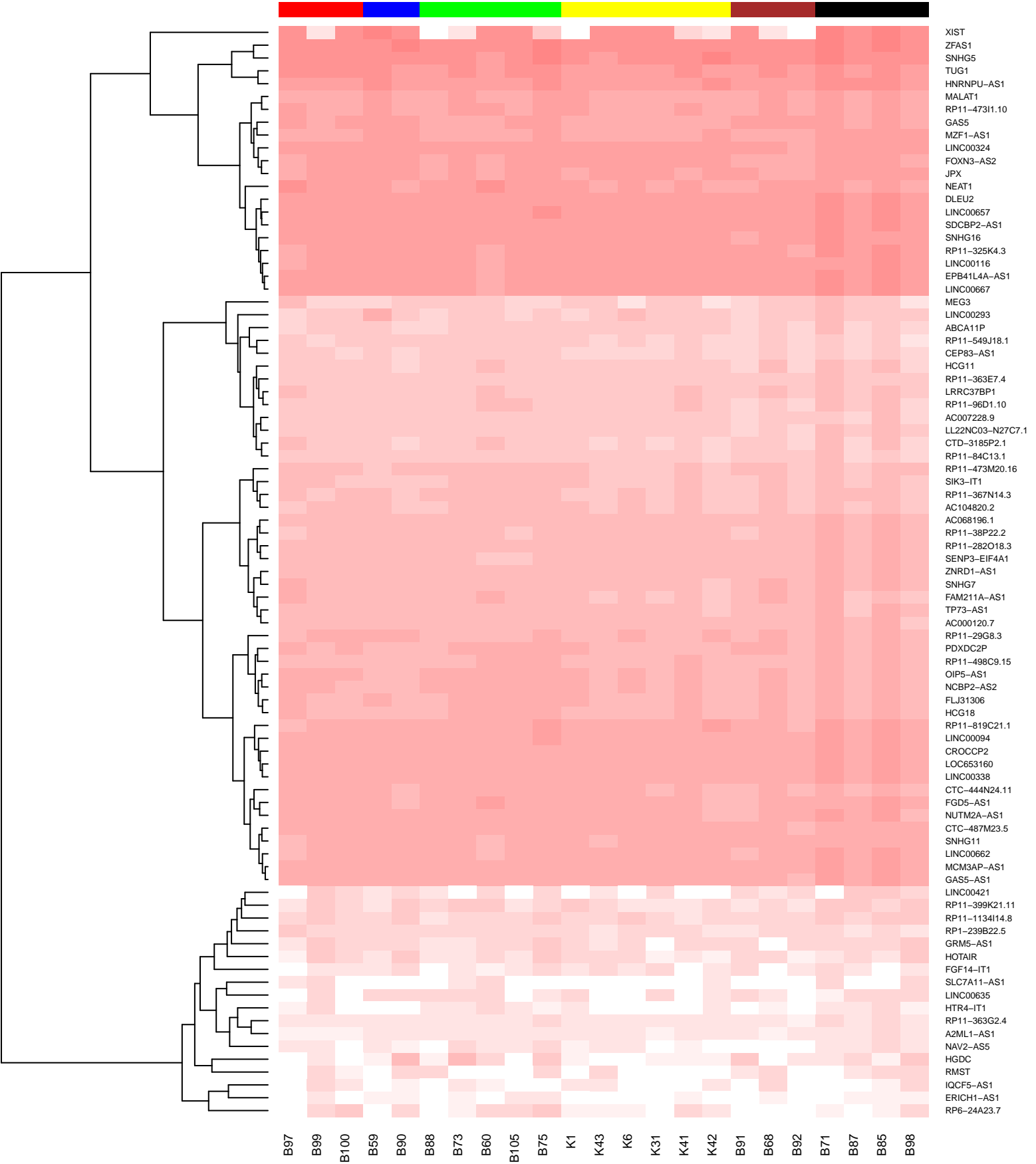

Supplement: Supplementary file 2 — Additional file 2: Heatmap of the relative expression of the lncRNAs in each sample of the discovery cohort. Color codes above the heatmap: blue: severe allergic asthma; red: mild allergic asthma; green: COPD; yellow: control; brown: non-allergic mild asthma; black: non-allergic severe asthma. [file 12967_2020_2581_MOESM2_ESM.pdf]

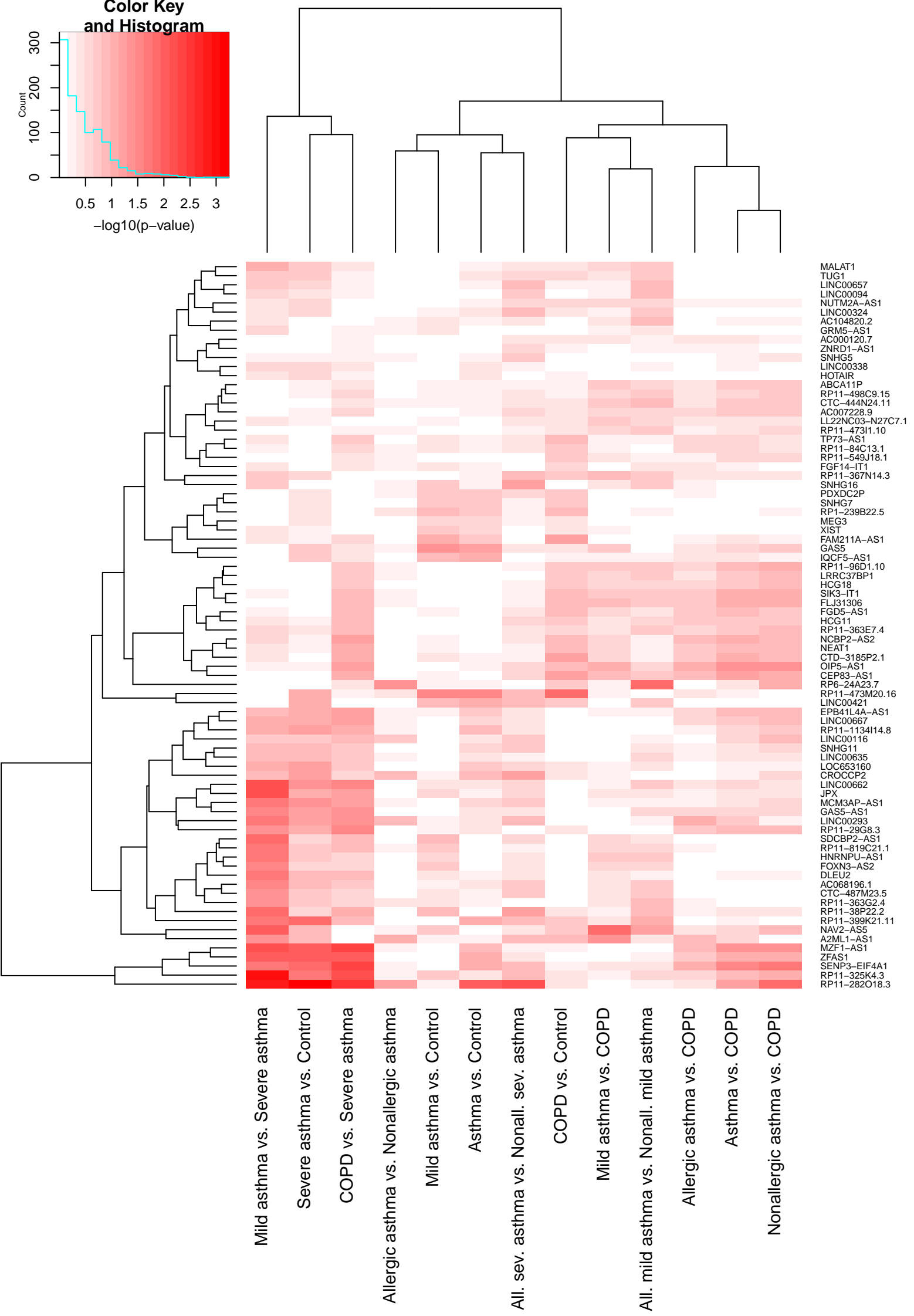

Supplement: Supplementary file 4 — Additional file 4: Heatmap of the sex adjusted –log10P values in comparison of the blood expression of 84 lncRNAs of the study subjects in the discovery cohort. [file 12967_2020_2581_MOESM4_ESM.pdf]

Color Key  
and Histogram

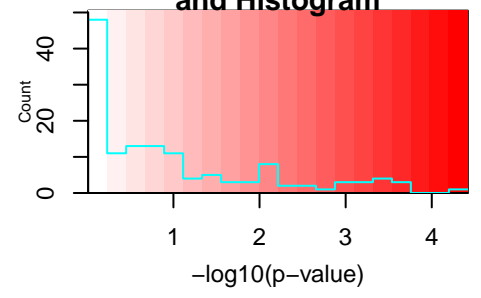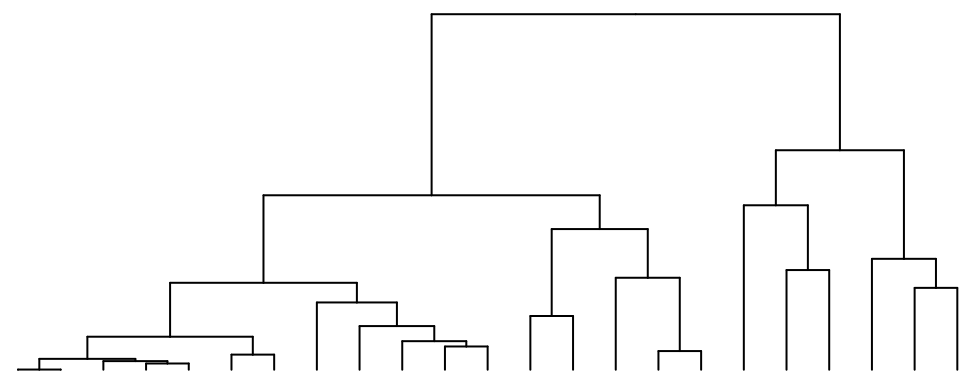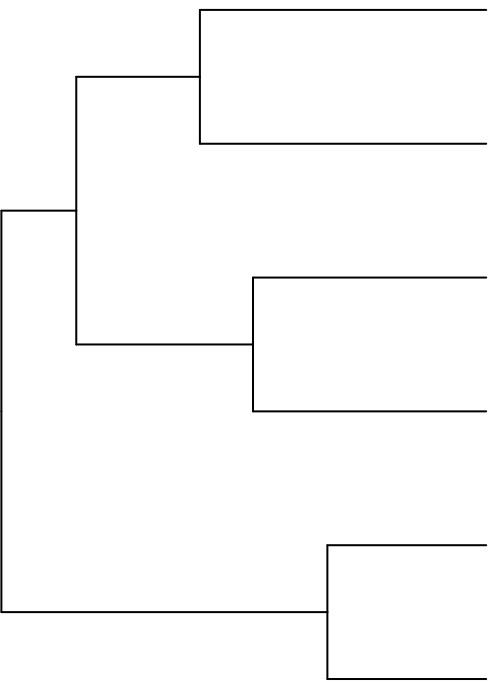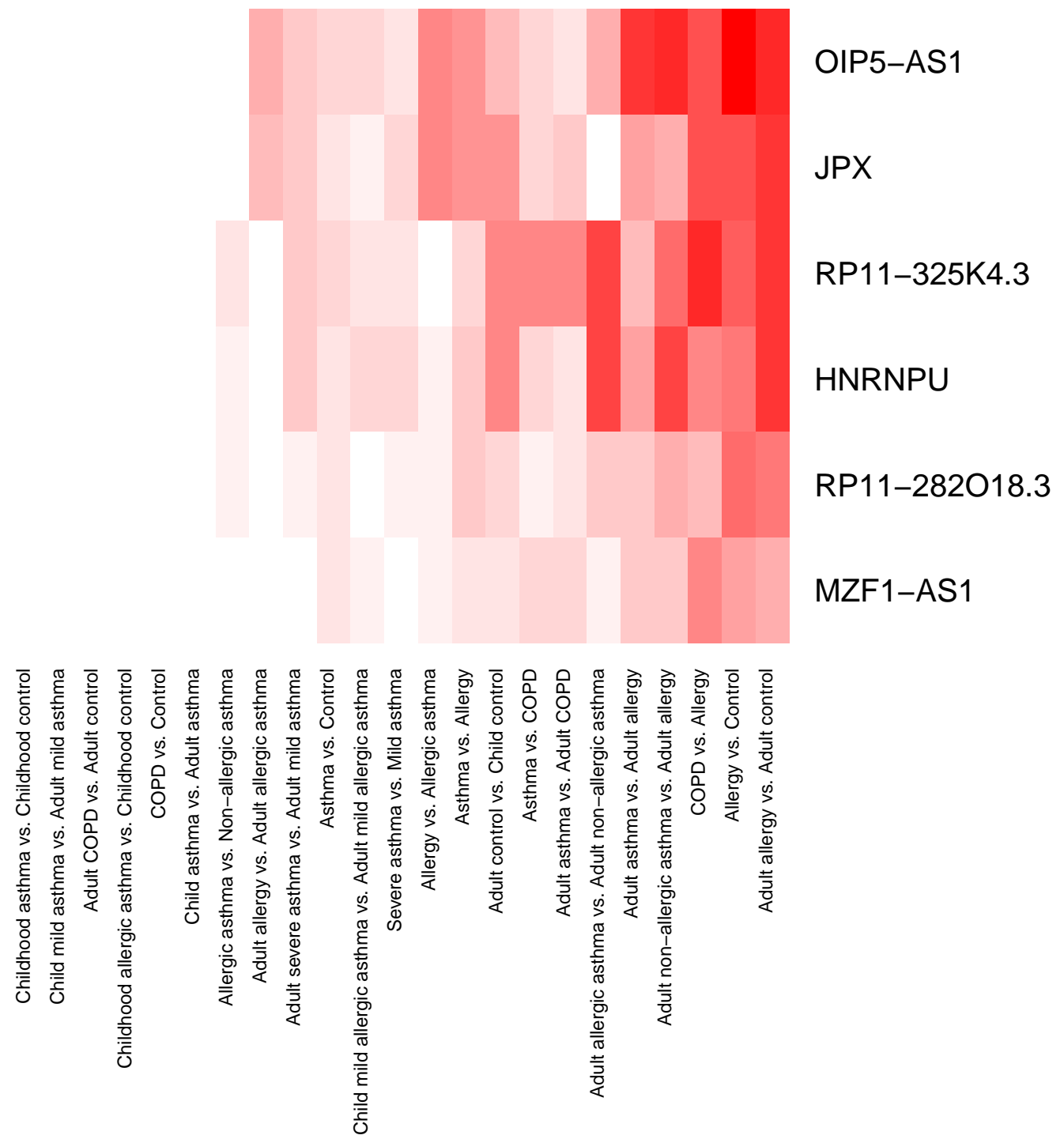

Supplement: Supplementary file 7 — Additional file 7: Heatmap of the adjusted –log10P values in comparison of the blood expression of the 6 selected lncRNAs of the study subjects in the replication cohort. [file 12967_2020_2581_MOESM7_ESM.pdf]

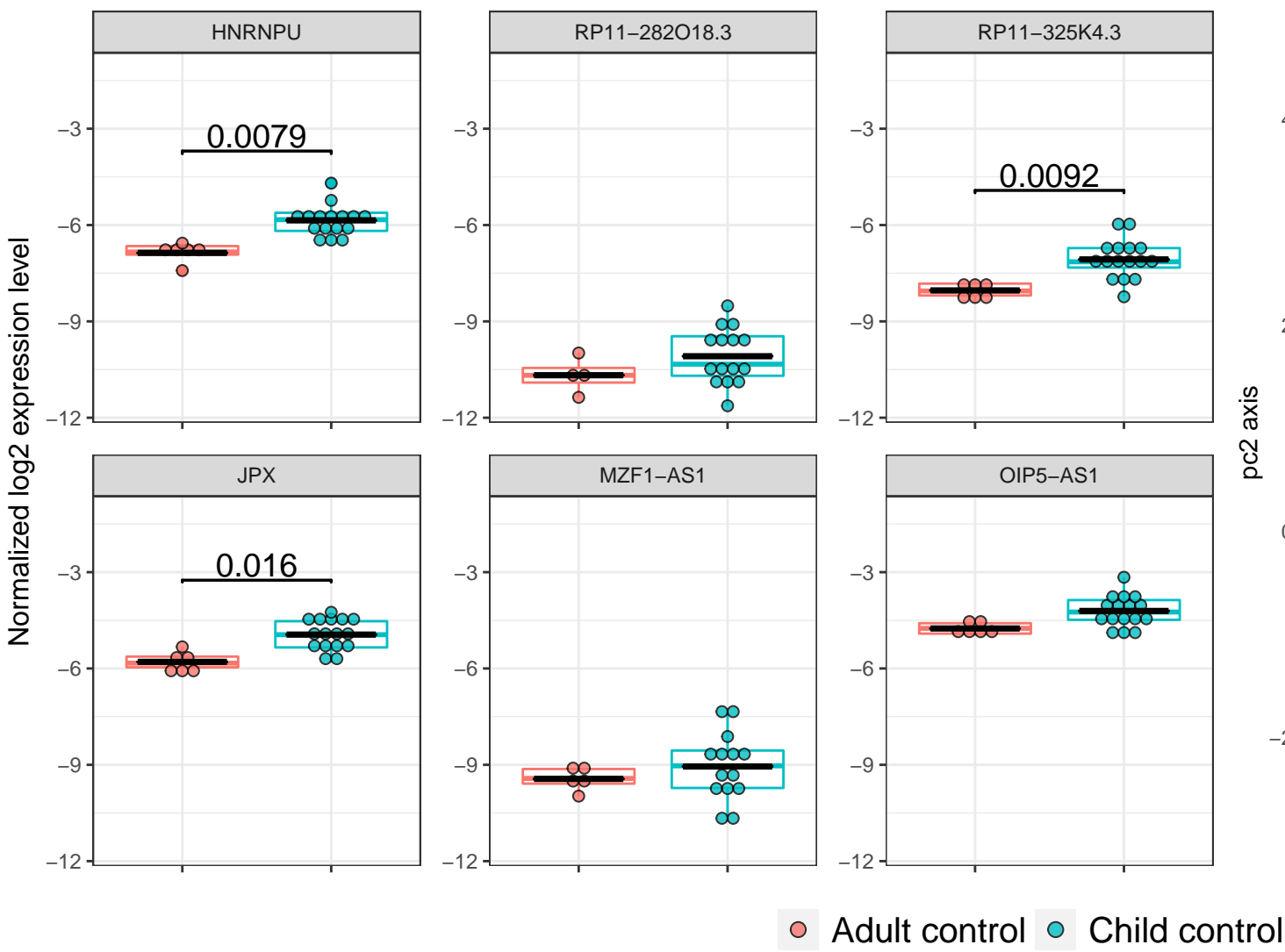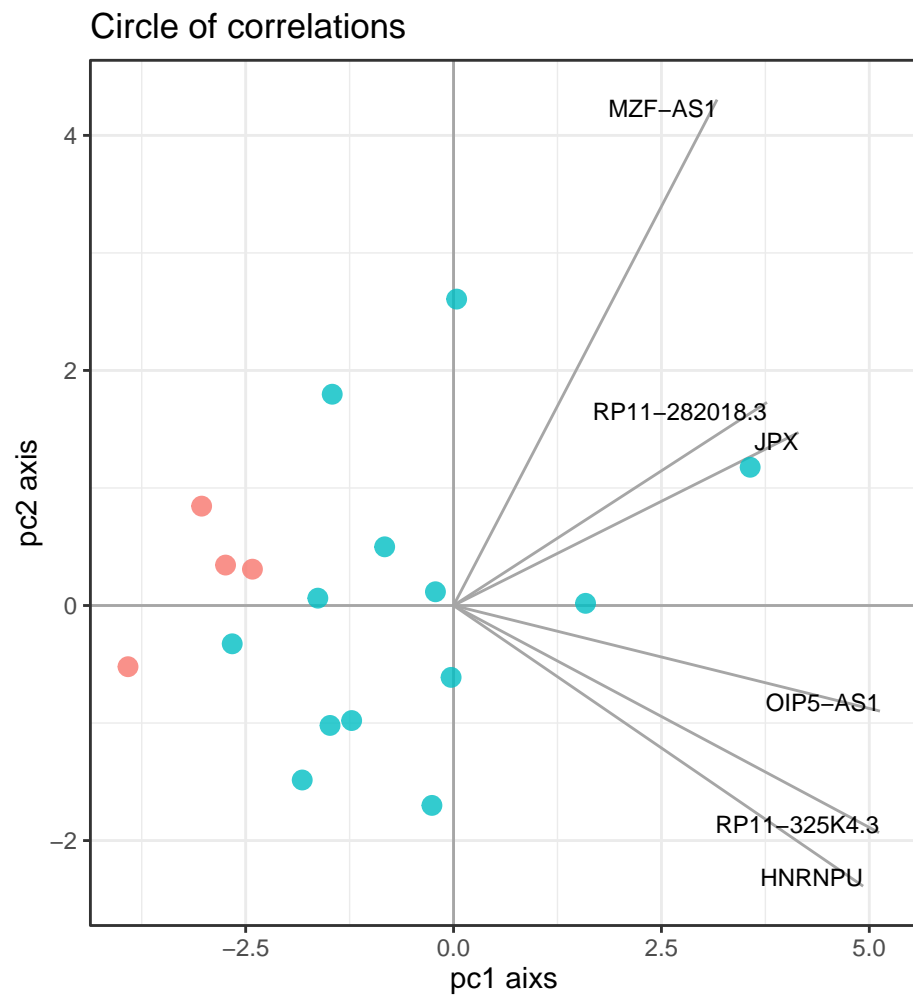

Supplement: Supplementary file 8 — Additional file 8: Comparison of the selected lncRNAs. Left: Comparison of the blood expressions of the 6 selected lncRNAs between adult and childhood controls. The adjusted P values are given for each comparison. Right: Principal component analysis bi-plot showing the scores of the samples (colored circles) and the loadings of the variables (i.e. the six selected lncRNA as grey arrows) along the first two principal components. [file 12967_2020_2581_MOESM8_ESM.pdf]

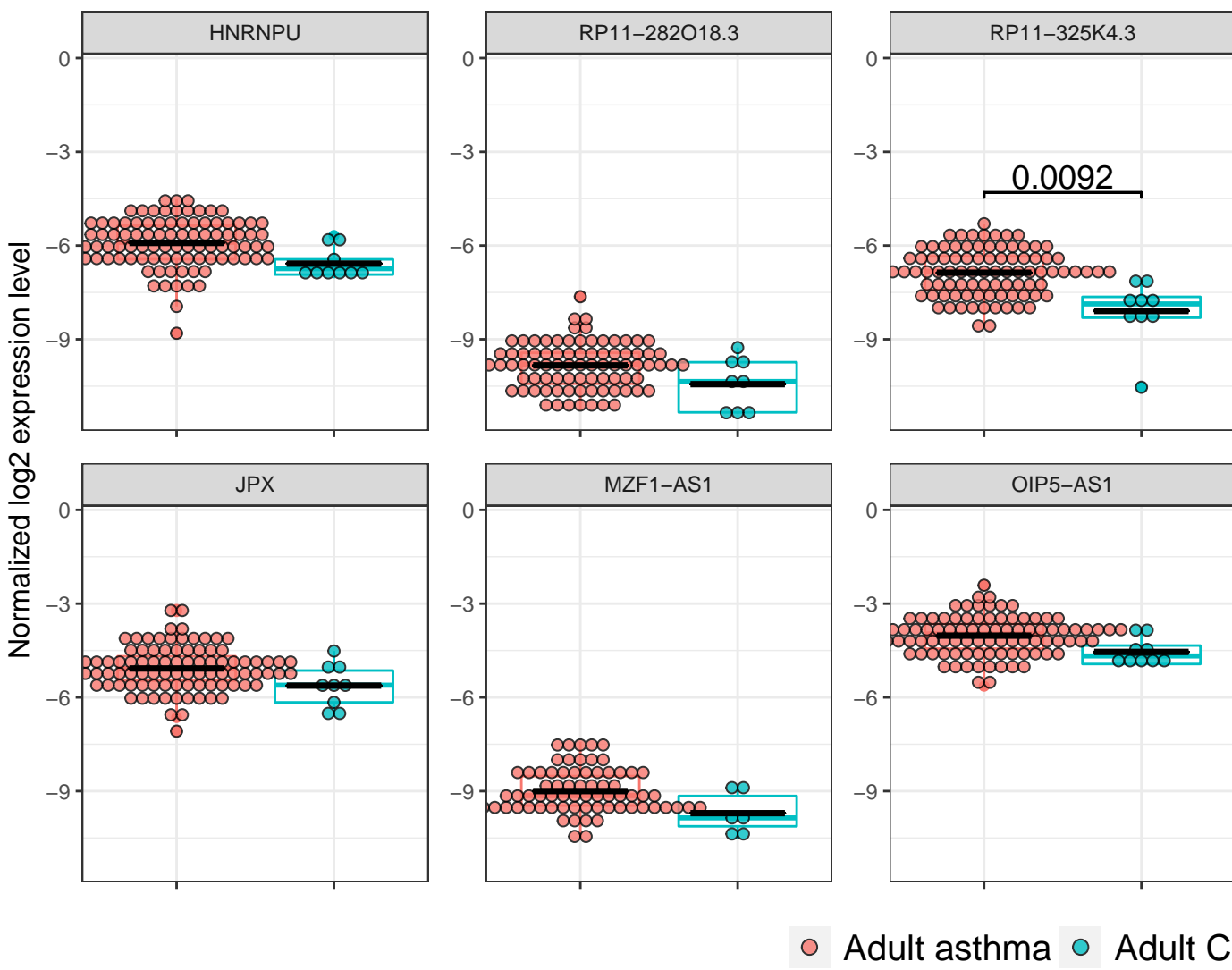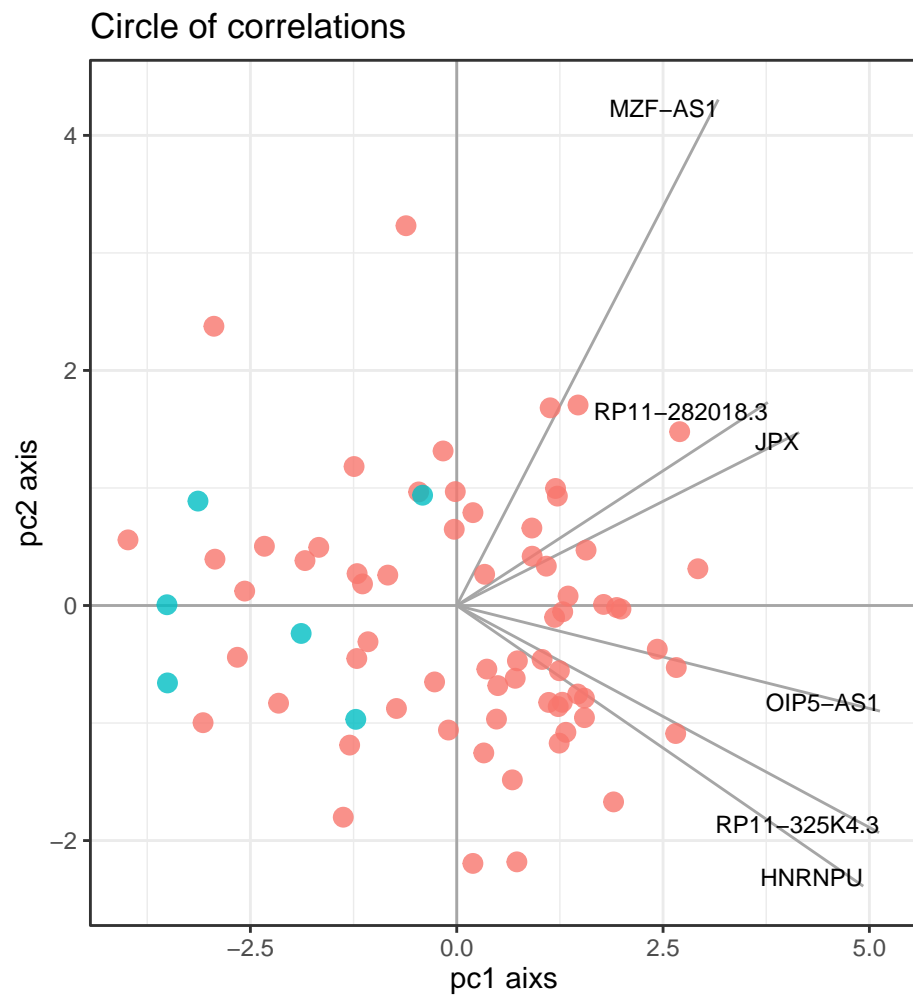

Supplement: Supplementary file 9 — Additional file 9: Comparison of the selected lncRNAs. Left: Comparison of the blood expression of the 6 selected lncRNAs between groups. The adjusted P-values are given for each comparison. Only those comparisons are depicted, where at least one significant difference was found. Right: Principal component analysis bi-plot showing the scores of the samples (colored circles) and the loadings of the variables (i.e. the six selected lncRNA as grey arrows) along the first two principal components. [file 12967_2020_2581_MOESM9_ESM.pdf]

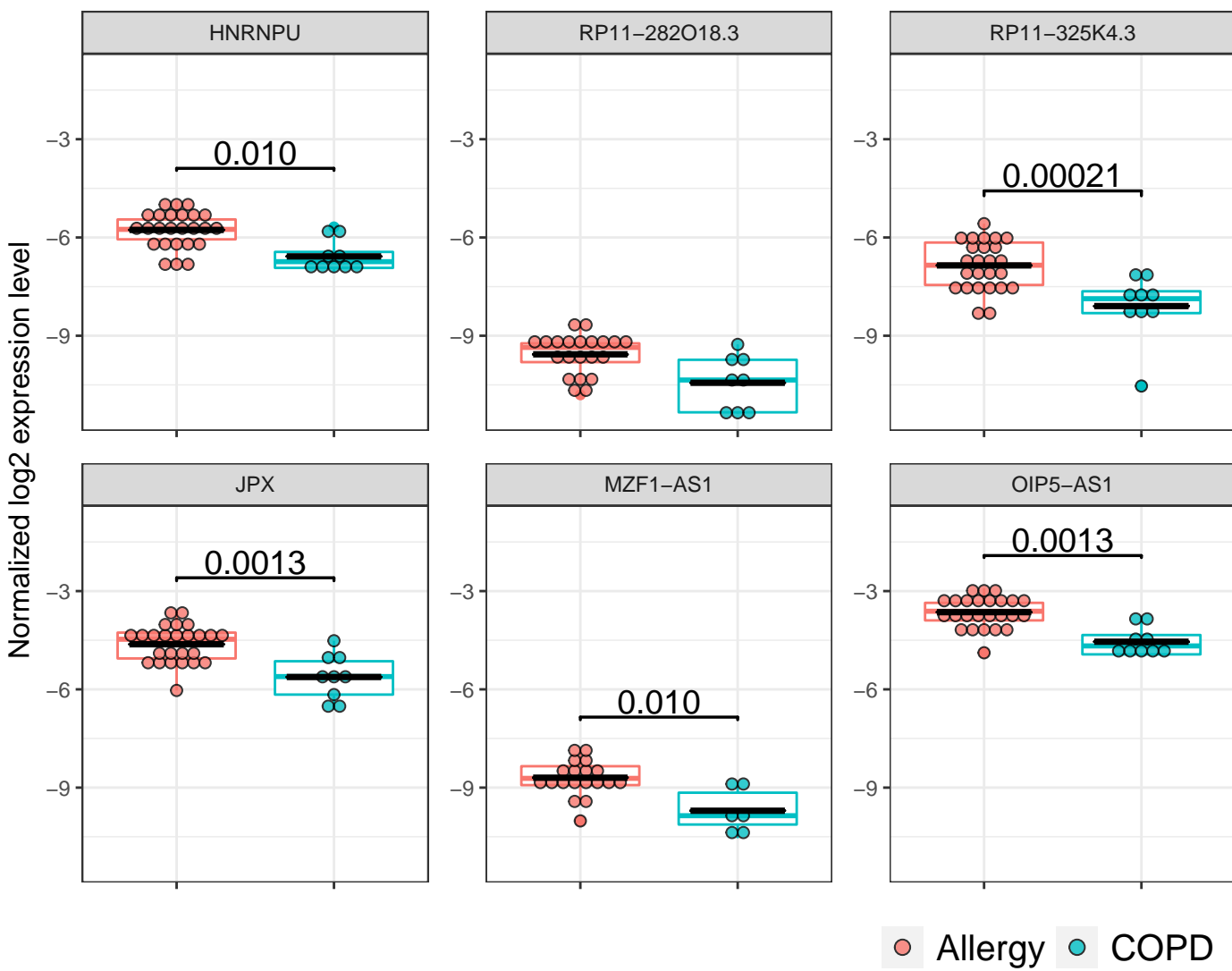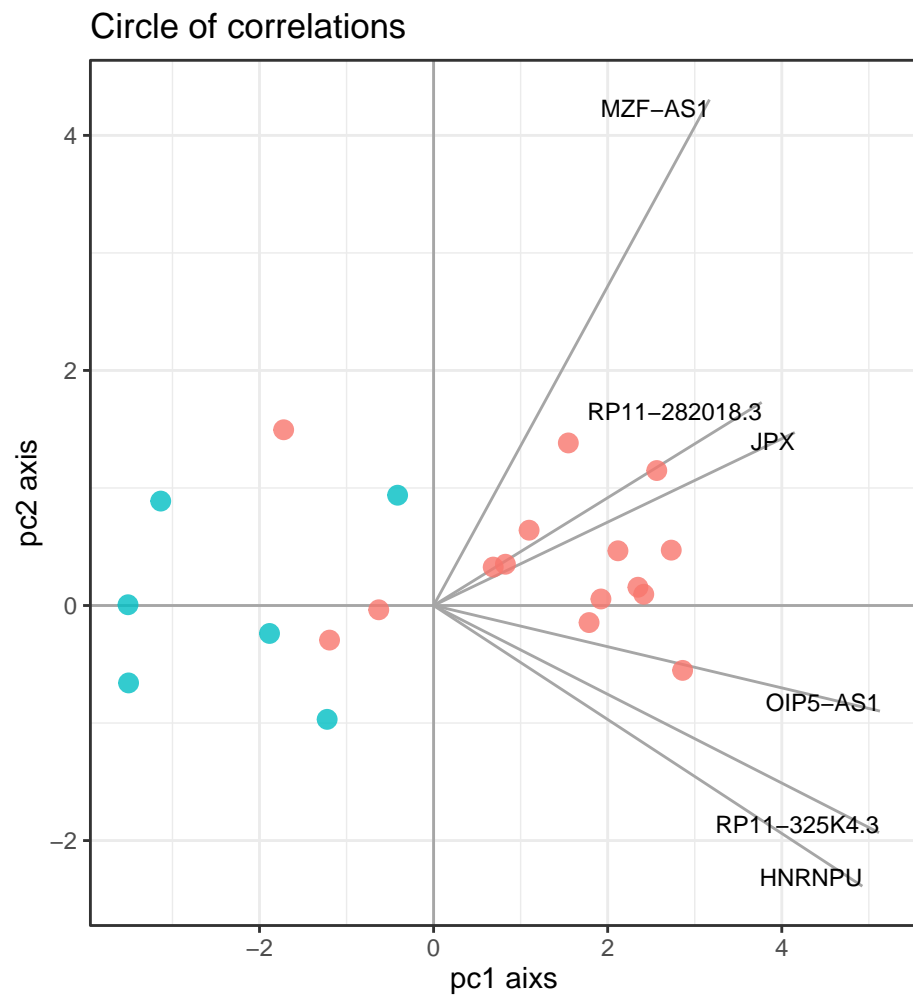

Supplement: Supplementary file 10 — Additional file 10: Comparison of the selected lncRNAs. Left: Comparison of the blood expression of the 6 selected lncRNAs between groups. The adjusted P-values are given for each comparison. Only those comparisons are depicted, where at least one significant difference was found. Right: Principal component analysis bi-plot showing the scores of the samples (colored circles) and the loadings of the variables (i.e. the six selected lncRNA as grey arrows) along the first two principal components. [file 12967_2020_2581_MOESM10_ESM.pdf]

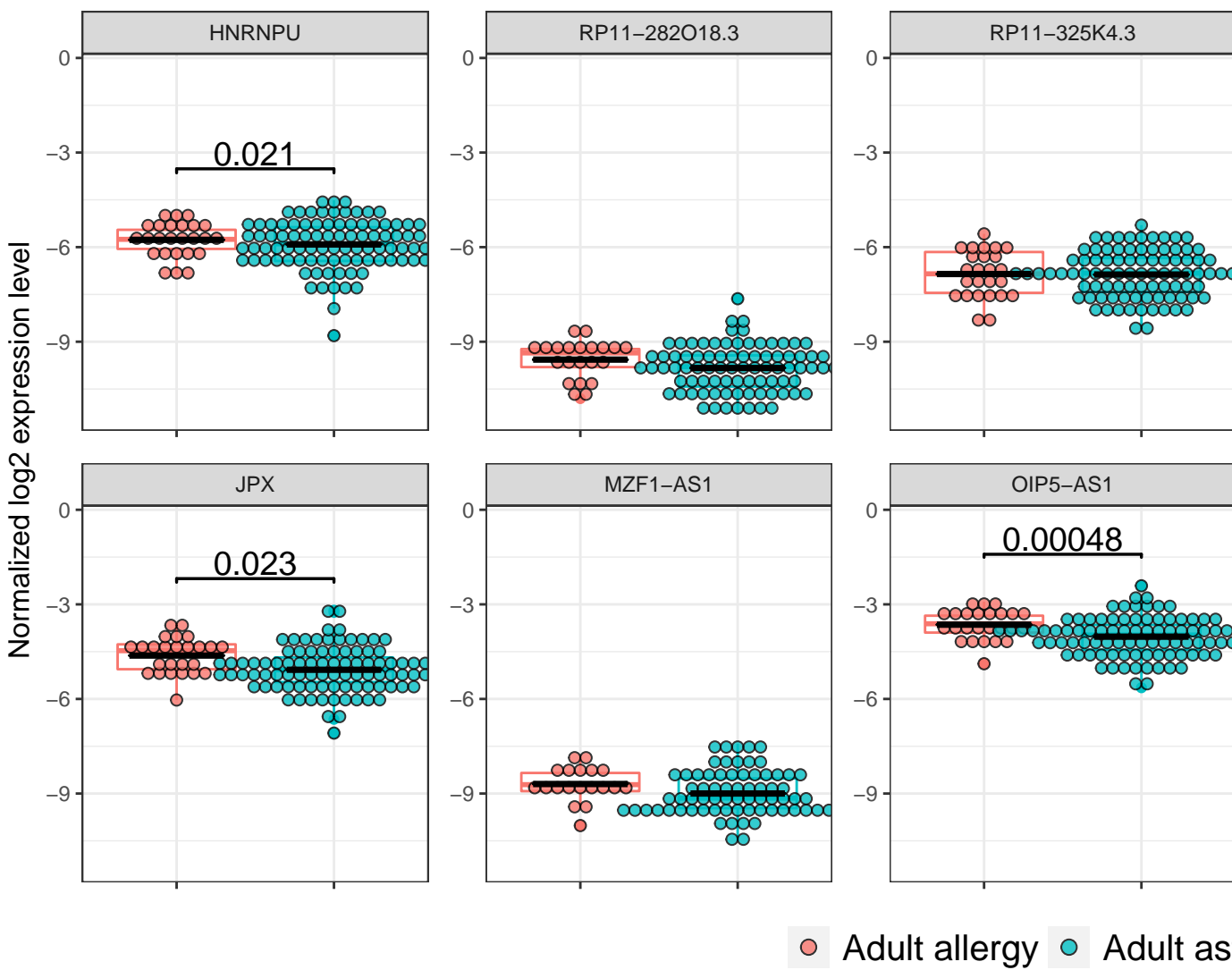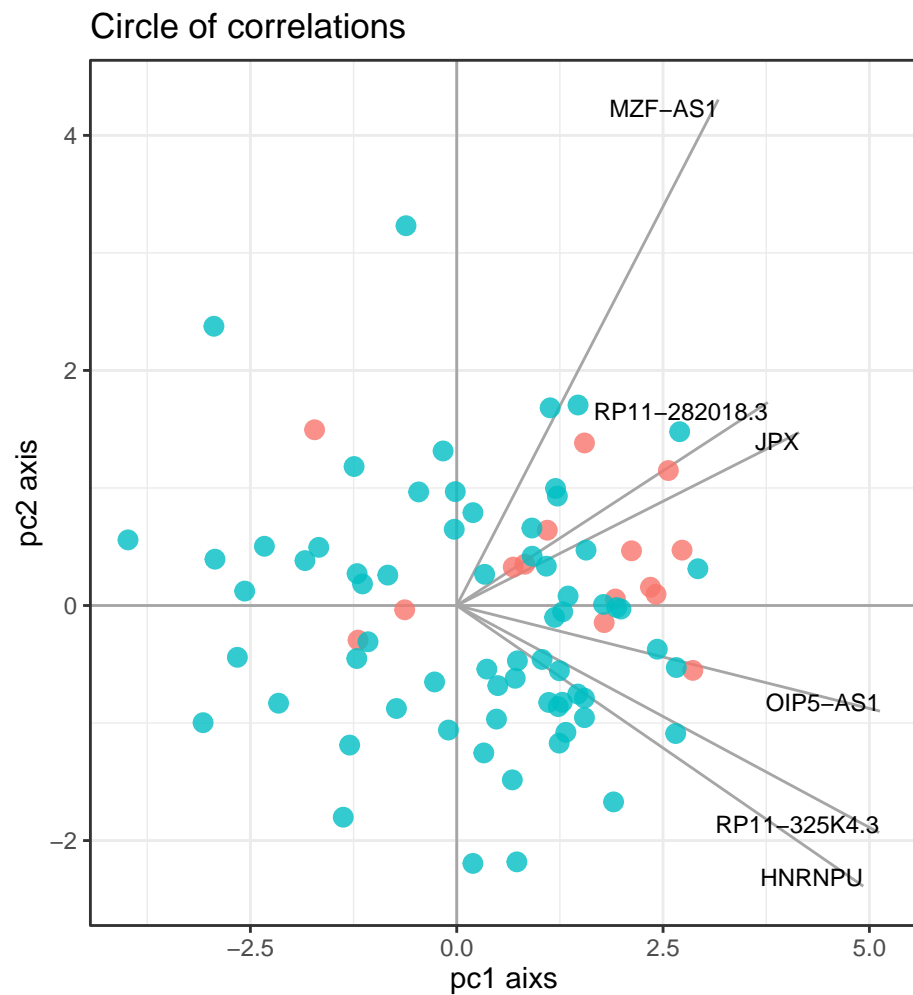

Supplement: Supplementary file 11 — Additional file 11: Comparison of the selected lncRNAs. Left: Comparison of the blood expression of the 6 selected lncRNAs between groups. The adjusted P-values are given for each comparison. Only those comparisons are depicted, where at least one significant difference was found. Right: Principal component analysis bi-plot showing the scores of the samples (colored circles) and the loadings of the variables (i.e. the six selected lncRNA as grey arrows) along the first two principal components. [file 12967_2020_2581_MOESM11_ESM.pdf]

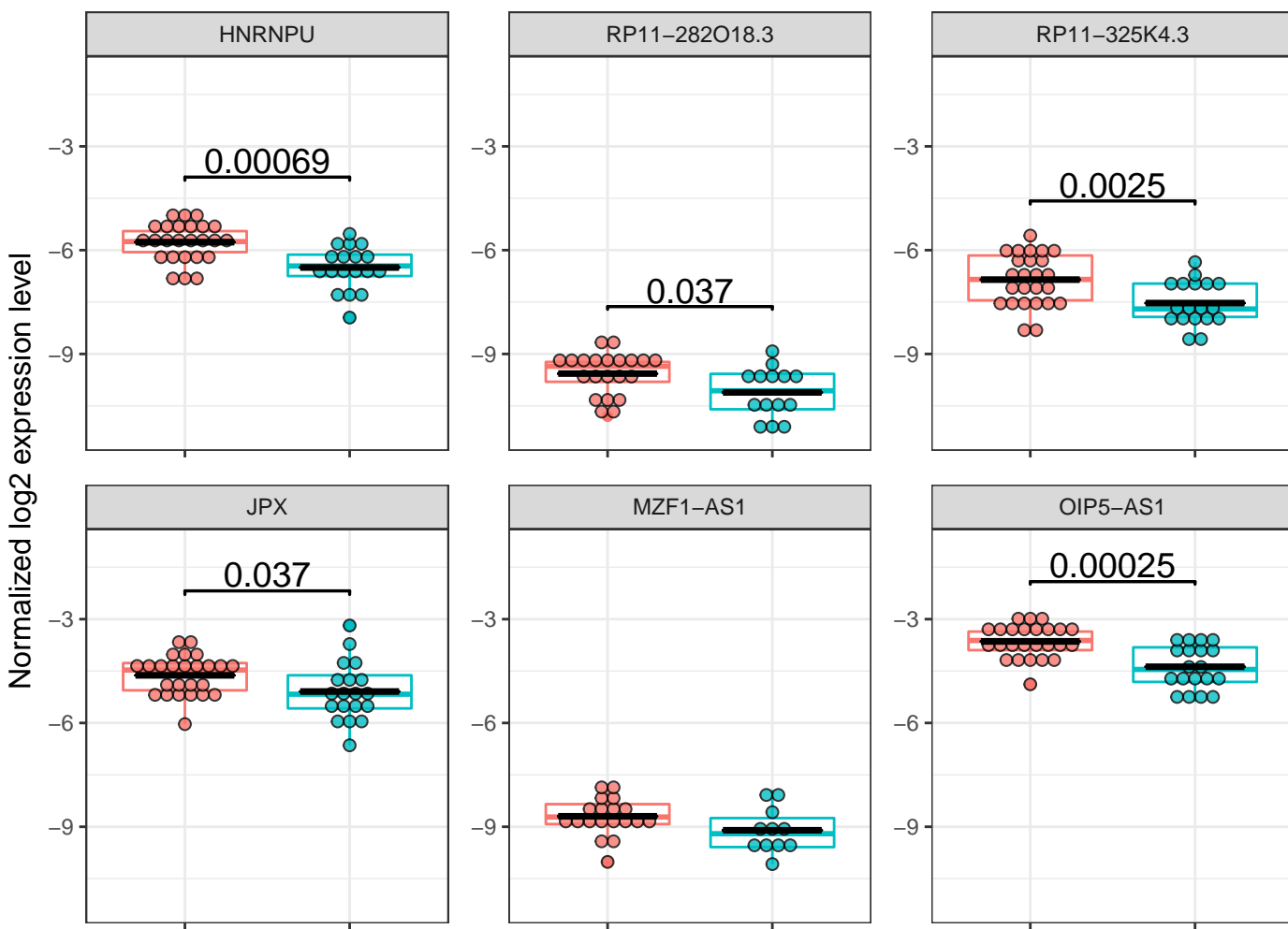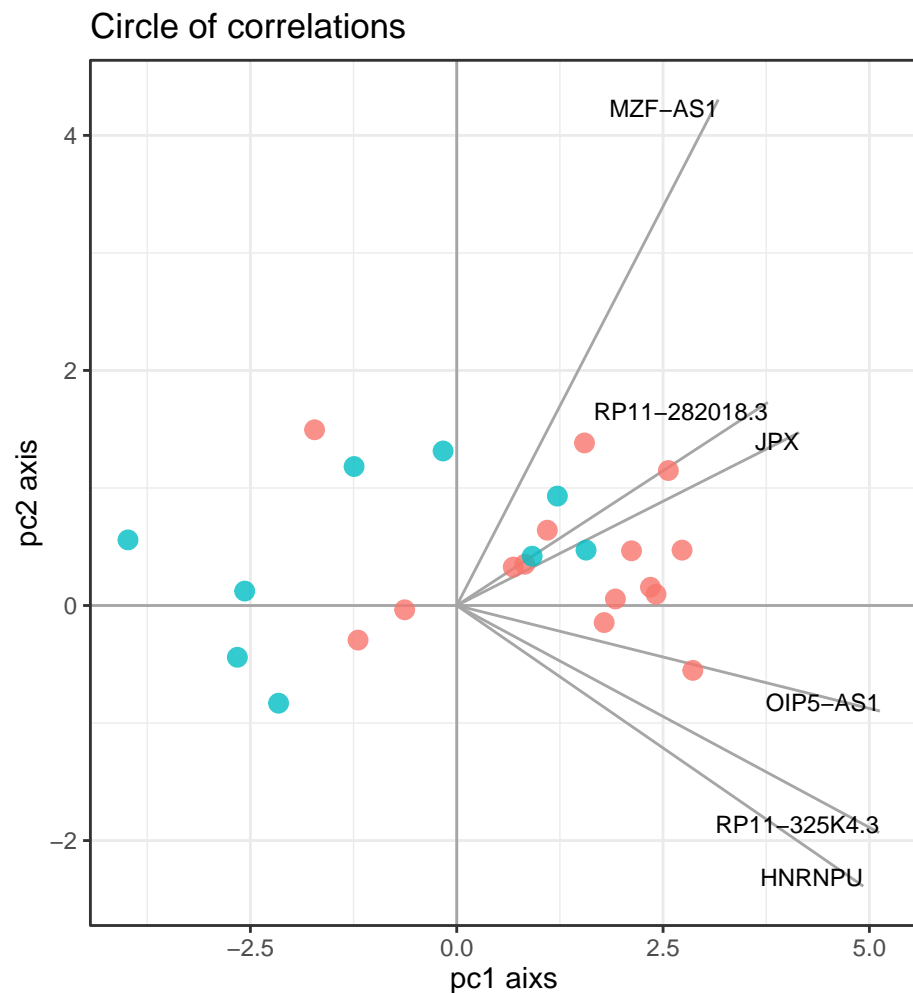

● Adult allergy ● Adult non-allergic asthma

Supplement: Supplementary file 12 — Additional file 12: Comparison of the selected lncRNAs. Left: Comparison of the blood expression of the 6 selected lncRNAs between groups. The adjusted P-values are given for each comparison. Only those comparisons are depicted, where at least one significant difference was found. Right: Principal component analysis bi-plot showing the scores of the samples (colored circles) and the loadings of the variables (i.e. the six selected lncRNA as grey arrows) along the first two principal components. [file 12967_2020_2581_MOESM12_ESM.pdf]

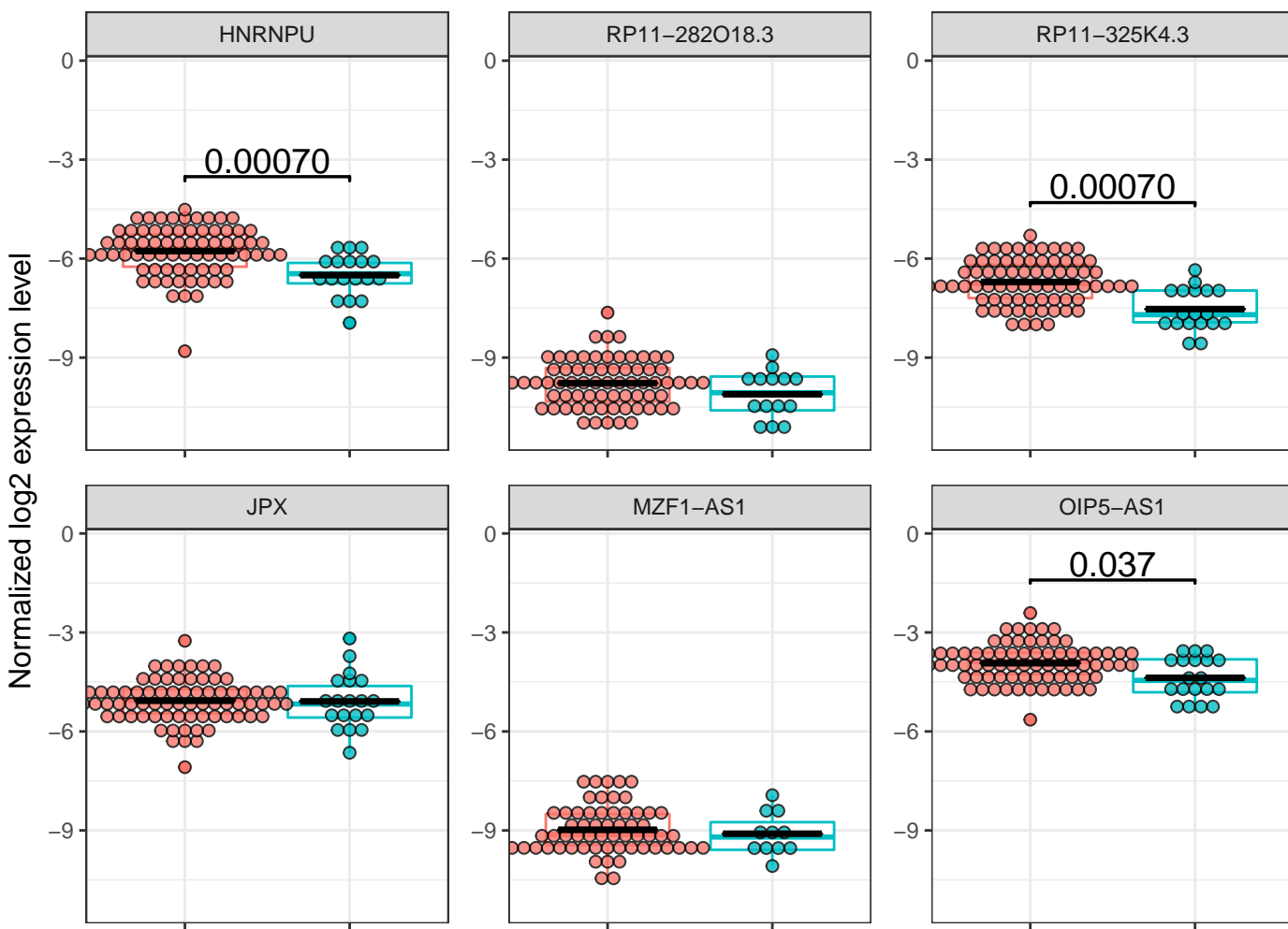

● Adult allergic asthma ● Adult non-allergic asthma

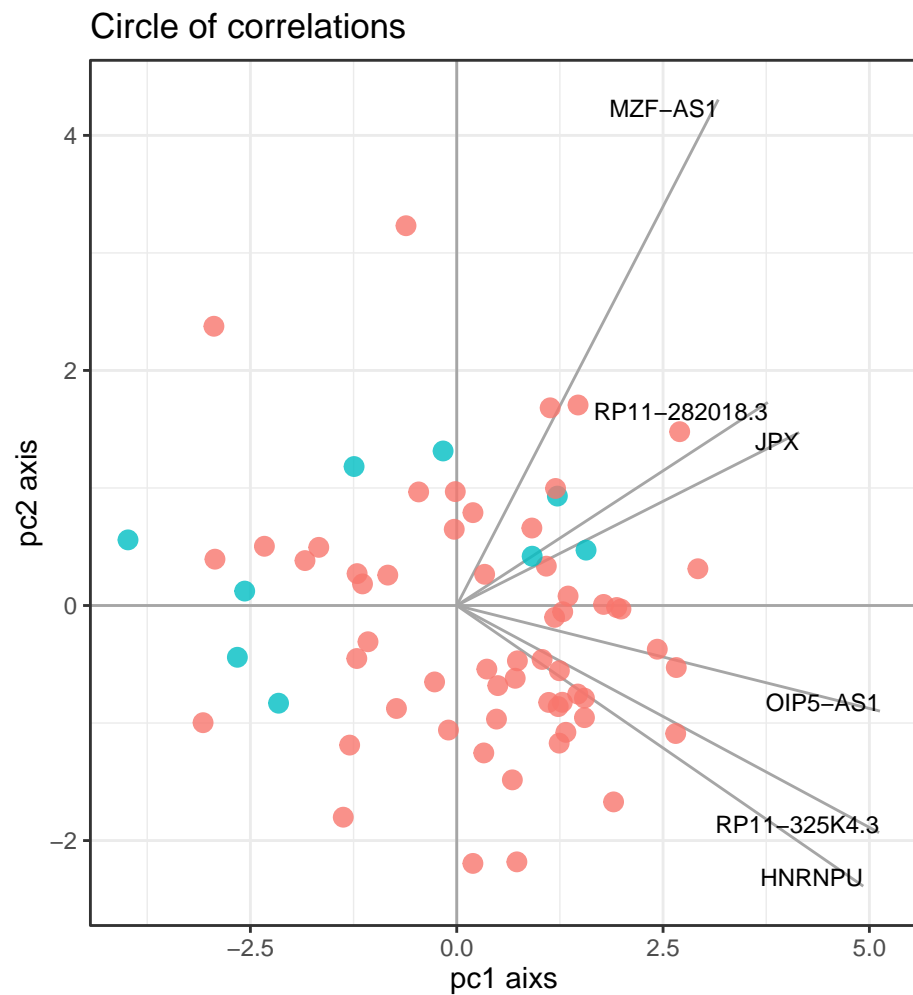

Supplement: Supplementary file 13 — Additional file 13: Comparison of the selected lncRNAs. Left: Comparison of the blood expression of the 6 selected lncRNAs between groups. The adjusted P-values are given for each comparison. Only those comparisons are depicted, where at least one significant difference was found. Right: Principal component analysis bi-plot showing the scores of the samples (colored circles) and the loadings of the variables (i.e. the six selected lncRNA as grey arrows) along the first two principal components. [file 12967_2020_2581_MOESM13_ESM.pdf]

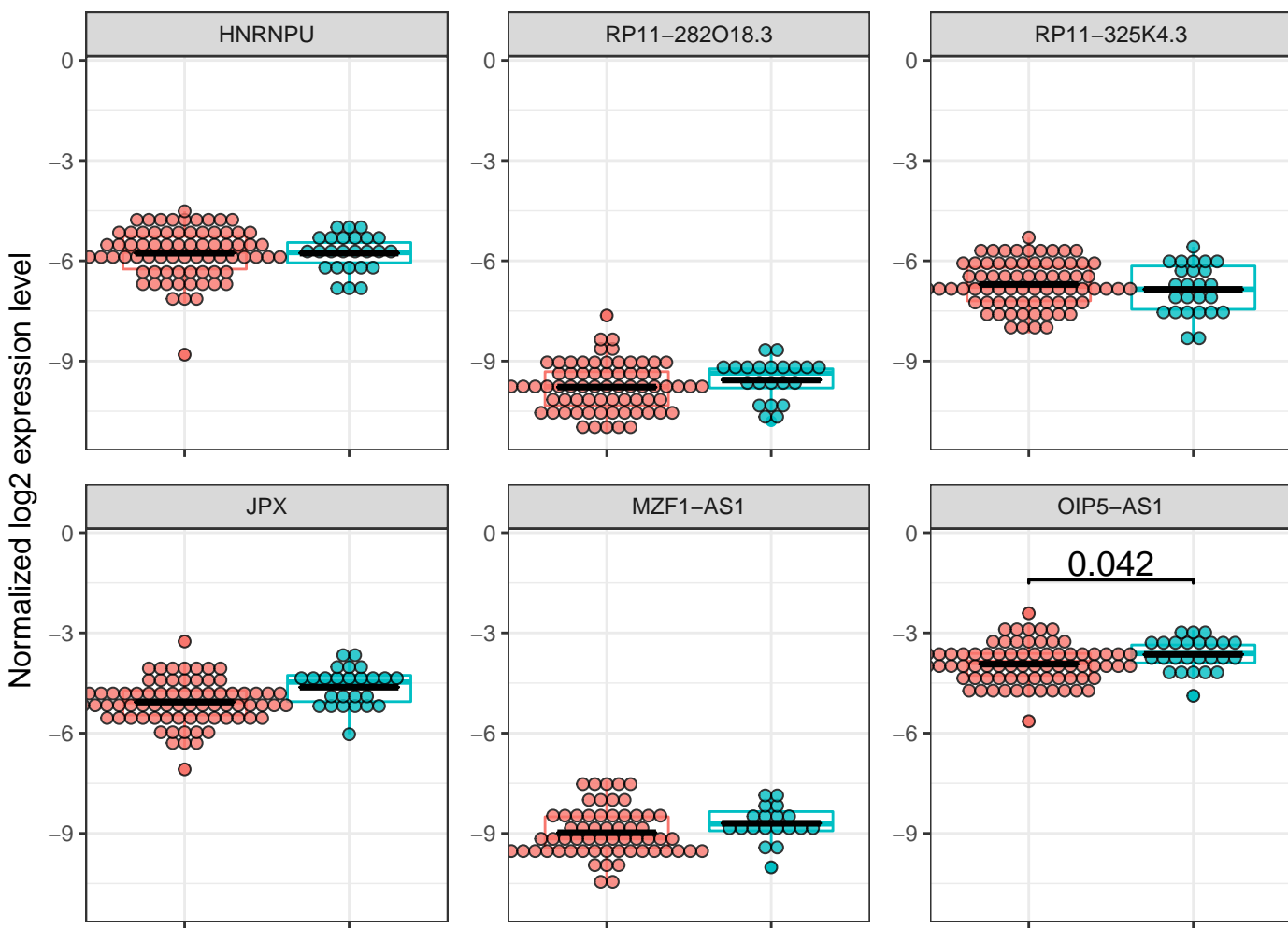

● Adult allergic asthma ● Adult allergy

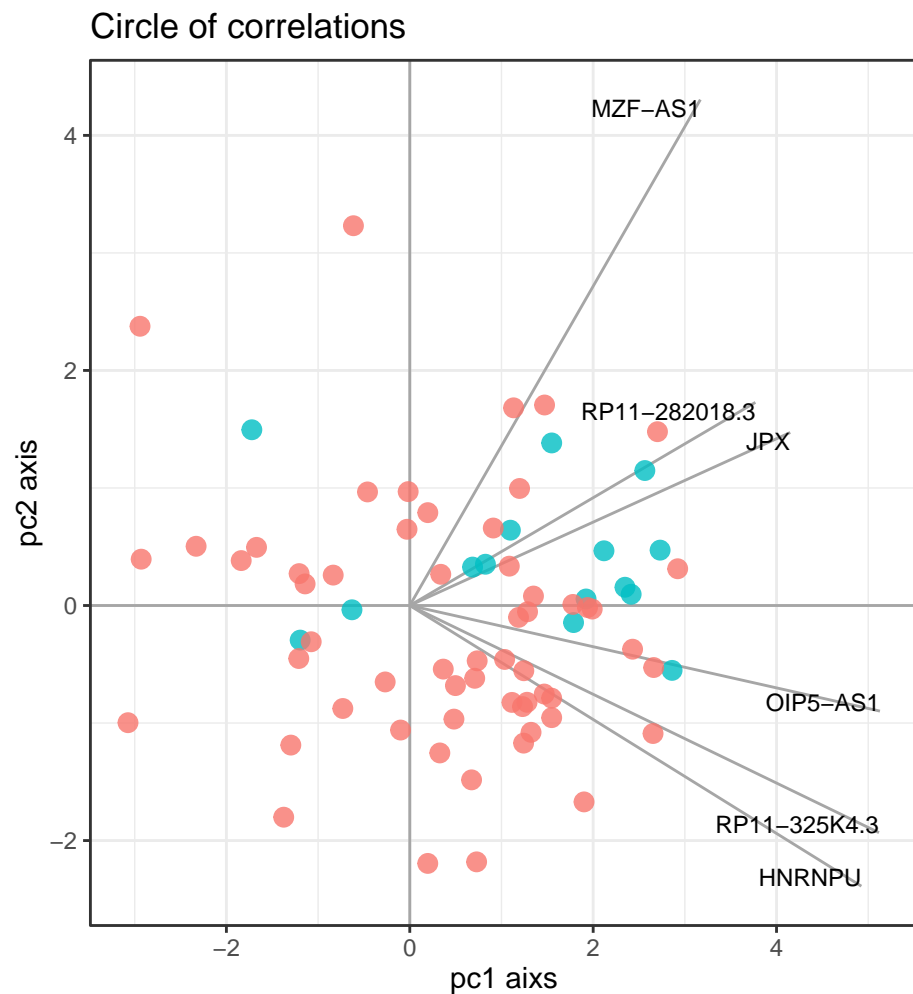

Supplement: Supplementary file 14 — Additional file 14: Comparison of the selected lncRNAs. Left: Comparison of the blood expression of the 6 selected lncRNAs between groups. The adjusted P-values are given for each comparison. Only those comparisons are depicted, where at least one significant difference was found. Right: Principal component analysis bi-plot showing the scores of the samples (colored circles) and the loadings of the variables (i.e. the six selected lncRNA as grey arrows) along the first two principal components. [file 12967_2020_2581_MOESM14_ESM.pdf]
