# Supplementary material for: Investigation of circulating lncRNAs as potential biomarkers in chronic respiratory diseases
Source: J Transl Med. 2020 Nov 10;18:422. doi: 10.1186/s12967-020-02581-9 (PMC7653503; doi:10.1186/s12967-020-02581-9)
Supplement: Supplementary file 3 — Additional file 3: Heatmap of the log2FC values in comparison of the blood expression of 84 lncRNAs of the study subjects in the discovery cohort. [file 12967_2020_2581_MOESM3_ESM.pdf]

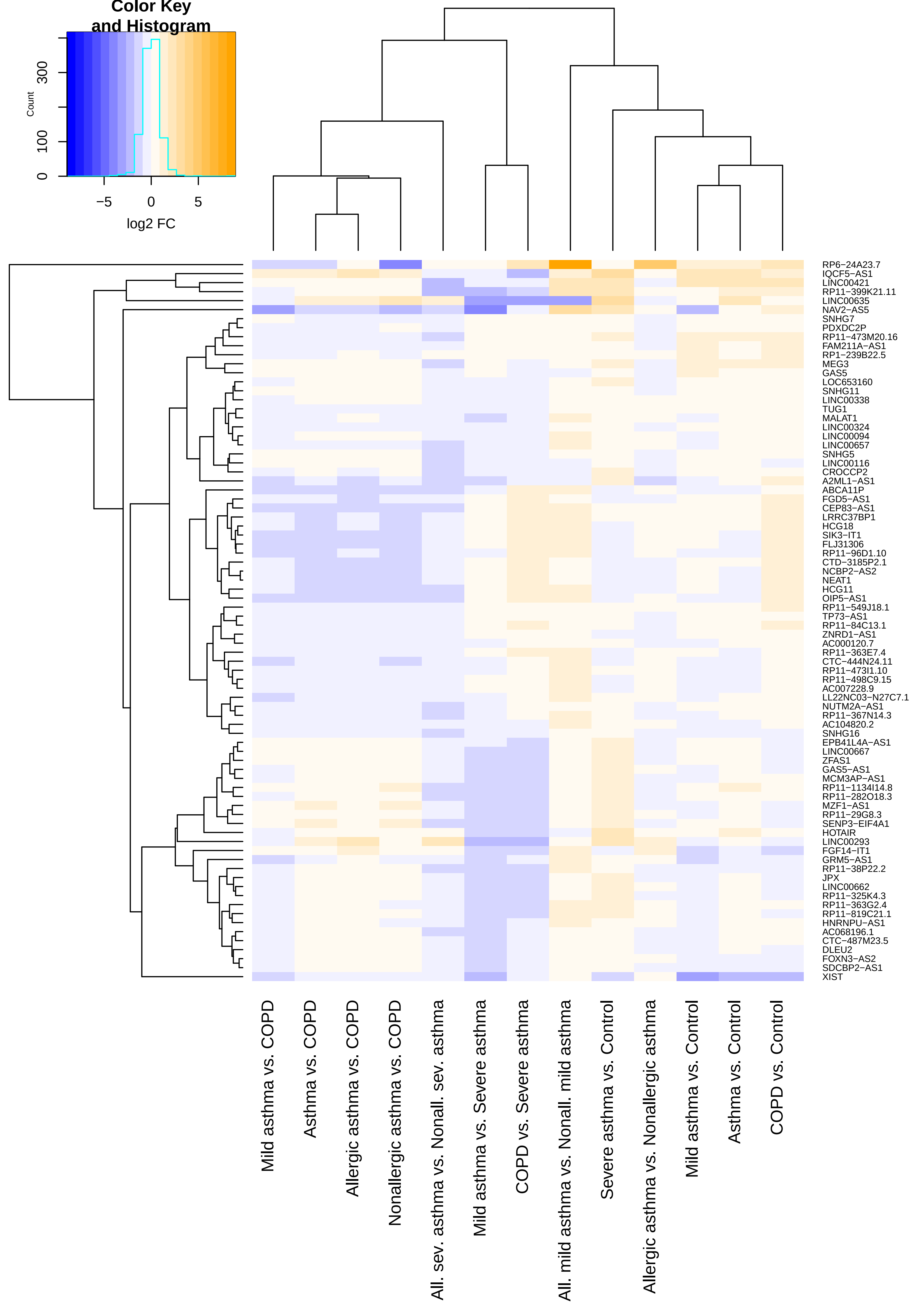

Color Key
and Histogram
Count
0
100
300
−5
0
5
log2 FC
RP6−24A23.7
IQCF5−AS1
LINC00421
RP11−399K21.11
LINC00635
NAV2−AS5
SNHG7
PDXDC2P
RP11−473M20.16
FAM211A−AS1
RP1−239B22.5
MEG3
GAS5
LOC653160
SNHG11
LINC00338
TUG1
MALAT1
LINC00324
LINC00094
LINC00657
SNHG5
LINC00116
CROCCP2
A2ML1−AS1
ABCA11P
FGD5−AS1
CEP83−AS1
LRRC37BP1
HCG18
SIK3−IT1
FLJ31306
RP11−96D1.10
CTD−3185P2.1
NCBP2−AS2
NEAT1
HCG11
OIP5−AS1
RP11−549J18.1
TP73−AS1
RP11−84C13.1
ZNRD1−AS1
AC000120.7
RP11−363E7.4
CTC−444N24.11
RP11−473I1.10
RP11−498C9.15
AC007228.9
LL22NC03−N27C7.1
NUTM2A−AS1
RP11−367N14.3
AC104820.2
SNHG16
EPB41L4A−AS1
LINC00667
ZFAS1
GAS5−AS1
MCM3AP−AS1
RP11−1134I14.8
RP11−282O18.3
MZF1−AS1
RP11−29G8.3
SENP3−EIF4A1
HOTAIR
LINC00293
FGF14−IT1
GRM5−AS1
RP11−38P22.2
JPX
LINC00662
RP11−325K4.3
RP11−363G2.4
RP11−819C21.1
HNRNPU−AS1
AC068196.1
CTC−487M23.5
DLEU2
FOXN3−AS2
SDCBP2−AS1
XIST
Mild asthma vs. COPD
Asthma vs. COPD
Allergic asthma vs. COPD
Nonallergic asthma vs. COPD
All. sev. asthma vs. Nonall. sev. asthma
Mild asthma vs. Severe asthma
COPD vs. Severe asthma
All. mild asthma vs. Nonall. mild asthma
Severe asthma vs. Control
Allergic asthma vs. Nonallergic asthma
Mild asthma vs. Control
Asthma vs. Control
COPD vs. Control
