# Supplementary material for: Investigation of circulating lncRNAs as potential biomarkers in chronic respiratory diseases
Source: J Transl Med. 2020 Nov 10;18:422. doi: 10.1186/s12967-020-02581-9 (PMC7653503; doi:10.1186/s12967-020-02581-9)
Supplement: Supplementary file 15 — Additional file 15. Detailed description of the systems biology analysis used for the prediction of lncRNA functions [file 12967_2020_2581_MOESM15_ESM.docx]

**Supplementary Material**

**Investigation of circulating lncRNAs as potential biomarkers in chronic respiratory diseases**

**Authors**

Zsófia Gál*^1^, András Gézsi*^2,3^, Ágnes F Semsei^1^, Adrienne Nagy^4^, Monika Sultész^4^, Zsuzsanna Csoma^5^, Lilla Tamási^6^, Gabriella Gálffy^7^, Csaba Szalai**^1,4^

^1^Department of Genetics, Cell- and Immunobiology, Semmelweis University, Budapest, Hungary

^2^Department of Measurements and Information Systems, Budapest University of Technology and Economics, Budapest, Hungary

^3^MTA-SE Immune-Proteogenomics Extracellular Vesicle Research Group, Semmelweis University, Budapest, Hungary

^4^Heim Pál Children’s Hospital, Budapest, Hungary

^5^National Korányi Institute of TB and Pulmonology, Budapest, Hungary

^6^Department of Pulmonology, Semmelweis University, Budapest, Hungary

^7^Pulmonology Hospital, Törökbálint, Hungary

*ZG and AG contributed equally to this work

****Correspondence:**

Csaba Szalai

Department of Genetics, Cell- and Immunobiology, Semmelweis University, Budapest, Hungary Email: [szalai.csaba@med.semmelweis-univ.hu](mailto:szalai.csaba@med.semmelweis-univ.hu); [szalaics@gmail.com](mailto:szalaics@gmail.com)

**Prediction of the putative functions of lncRNAs**

We performed a systems biology analysis to identify the putative functional pathways and Gene Ontology terms associated with each of the six lncRNAs (chosen for the replication cohort). The overview of this analysis can be seen in Supplementary Figure 1.

*Brief overview of the analysis*

First, we constructed a heterogeneous lncRNA-gene network with the combination of three biological networks, namely (1) the tissue specific transcriptional similarity of lncRNAs derived from the GTEx database, (2) experimentally validated lncRNA-target gene network derived from the LncTarD database and (3) highly confident protein-protein interaction data from the STRING database. The nodes in the combined network represent genes (lncRNAs and other coding and non-coding genes), and the edges represent functional connection between the corresponding nodes. Next, we applied a network propagation algorithm, namely random walk with restart, initiated from each of the six lncRNAs to quantitatively prioritize the genes that are expected to be regulated by the given lncRNA. Finally, based on the quantitatively prioritized target genes, we performed gene set enrichment analysis (GSEA) to identify the functionally relevant pathways and Gene Ontology terms.

*Details of the analysis*

As a first step, we constructed three different biological networks and then combined them into a heterogeneous lncRNA-gene network. The three networks were created as the following:

**1. Tissue-specific transcriptional similarity network.** We downloaded a whole blood tissue-specific gene regulatory network (created by Sonawane et al. [1]) that were predicted by the PANDA software [2] combining protein-protein interactions, transcription factor information (motifs) and co-expression data originating from the Genotype-Tissue Expression (GTEx) Project. This network is an undirected bipartite network connecting transcription factors (TFs) and genes. From this network we constructed a gene-gene similarity network for all possible gene pairs calculated as the cosine similarity of TF profiles (i.e. a binary vector indicating which TFs regulates a particular gene) and kept those edges that had cosine similarity score greater than 0.3. A cosine similarity score of 1 means that two genes are regulated by exactly the same TFs, and a score of 0 means that two genes have no common TFs. This process resulted in a weighted sparse network that reflects whole blood tissue-specific transcriptional similarity of genes.

**2. Experimentally validated lncRNA-target gene network.** We downloaded the manually curated LncTarD database (version 1.0) [3] that contains experimentally-supported functional lncRNA-target regulations. We constructed an unweighted sparse network from this dataset by integrating those lncRNA-target gene pairs where both the lncRNA and the target gene could be mapped to the Ensembl database.

**3. Protein-protein interaction network.** We retrieved human protein-protein interaction data from the STRING database (version 10.5) [4] and filtered those interactions that had a confidence score greater than 0.7. Then, we mapped the proteins to the corresponding genes using the Ensembl BioMart tool. This resulted in an unweighted sparse gene-gene network.

The underlying assumptions of this method are that (1) lncRNAs that have a similar transcriptional profile (i.e. there is a high overlap between the transcriptional factors that are co-expressed with two different lncRNAs) are more likely to share their target genes, (2) lncRNAs and their target genes are more likely to have similar transcriptional profiles and (3) a gene that is interacting with many target genes of a given lncRNA is more likely to be regulated by that lncRNA. If any of these assumptions holds in case of an lncRNA-target gene pair, they will be closer to each other in the combined network than a random lncRNA-gene pair.

Next, we combined these three networks by the construction of a meta-network (see Supplementary Figure 1A). The meta-network consisted of two types of meta-nodes: lncRNAs (or more precisely lncRNA coding genes) and genes, respectively. Note, that the gene meta-node may also contain lncRNA coding genes. The meta-network consisted of four meta-edges, namely (1) the tissue-specific transcriptional similarity of lncRNAs, (2) the tissue-specific transcriptional similarity between lncRNAs and genes, (3) the experimentally validated lncRNA-target gene pairs connecting lncRNAs and genes, and (4) protein-protein interaction of genes. This meta-network induced a so called heterogeneous lncRNA-gene network (see Supplementary Figure 1B).

Afterwards, we applied the random walk with restart network propagation algorithm initiated from each of the six lncRNAs to quantitatively prioritize the genes that are expected to be functionally relevant with respect to a particular lncRNA (see Supplementary Figure 1C). The steady-state distribution of the random walk with restart process was computed with the following formula:

$$p_{\infty}=\alpha\left( I-\left( 1-\alpha\right)P^{T} \right)^{-1}*p_{0},$$

where $p_{0}$ is a vector that represents the initial state of the random walker (i.e. $p_{0}\left( v \right)=1$ for the query lncRNA node and $p_{0}\left( v \right)=0$ for all other nodes in the network), the parameters $\alpha$ reflects the probability of the random walker to return into the initial state, $I$ is the identity matrix, and $P$ is the state transition matrix defined by $P=D^{-1}A$, where $A$ is the adjacency matrix of the combined lncRNA-gene network, and $D$ is its degree matrix.

The parameter $\alpha$ describes the trade-off between prior information and network smoothing [5], and its value was set to 0.2 which gives relatively higher weight to the network (i.e. it lets the random walker to walk further away from the initial node), but according to simulations, the random walker will be located at most 3 steps away from the initial node with probability of more than 0.9. Note, however, that in accordance with other studies [6], [7] the results were not sensitive to the change of the $\alpha$ parameter.

Statistical significance of propagation scores were evaluated by permutation testing as follows: for a given lncRNA, the algorithm yielded an original score for each potential target gene; then we randomly selected 1000 lncRNAs and initiated the propagation method from those lncRNAs, which yielded another 1000 “random“ scores for each gene. Then the p-value of a gene was computed as the number of times a random score exceeded the original score of the gene. Genes were considered to be potential target genes when the p-value was below 0.05.

At a final step, we performed gene set enrichment analysis based on the quantitatively prioritized target genes to identify the functionally relevant pathways and Gene Ontology terms (see Supplementary Figure 1D). GSEA was conducted by the clusterProfiler R package [8]. The Gene Ontology and Reactome gene sets were retrieved from the MSigDB database (version 7.1) [9], and biological pathways from the WikiPathways database (version 20200510) [10] were also used. Gene sets were considered to be significant when the false discovery rate (FDR) value was below 0.1.

*Validation of the method*

We validated this computational method by evaluating the predictive performance of the lncRNA-target gene prediction step. For that we utilized the experimentally validated lncRNA-target gene information from the LncTarD database. Namely, we selected those lncRNAs that have at least five validated target genes, and are present in the Ensembl and GTEx databases, and predicted their target genes. Note, that as the LncTarD database was also used for constructing the combined network, we omitted the known lncRNA-target gene edges from the network when predicting the targets of a given lncRNA. Based on the predicted and the known targets we computed the area under the receiver operating characteristic curve (AUC) for each selected lncRNA, and we found that the accuracy of the method was generally high (mean AUC = 0.857, sd = 0.055, range = 0.687 - 0.973). AUC computations were conducted by the pROC R package [11].

*Earlier methods*

Several computational lncRNA function prediction methods have been proposed. Guo *et al.* [12] applied a global network-based strategy to annotate the functions of lncRNAs by integrating gene expression data and protein interaction data and using a network-based global function predictor. Linc2GO [13] was developed as a web resource to provide comprehensive functional annotations for human lincRNA, based on integrated microRNA-mRNA and microRNA-lincRNA interaction data. The LncRNAtor study [14] collected 208 RNA-Seq datasets to provide expression profiles in various tissues, diseases and developmental stages, and identified co-expressed mRNA of lncRNAs, which were then subjected to functional enrichment analysis such as Gene Ontology or KEGG pathways. LncRNA2Function [15] was also developed as a web application, that was based on expression correlation between lncRNAs and protein-coding genes across 19 human normal tissues, and used the hypergeometric test to functionally annotate lncRNAs with significantly enriched functional terms among the protein-coding genes that are significantly co-expressed with the lncRNAs. The most similar to our proposed method, the LncRNAs2Pathways tool [16] aims to identify the functional pathways influenced by the combinatorial effects of a set of interesting lncRNAs based on a global network propagation algorithm using an integrated network of co-expression and protein–protein interaction data. Regrettably, most of the methods/tools described above were not available at the time of our analysis.

**Reference**

[1] A. R. Sonawane *et al.*, “Understanding Tissue-Specific Gene Regulation,” *Cell Rep.*, vol. 21, no. 4, pp. 1077–1088, Oct. 2017.

[2] K. Glass, C. Huttenhower, J. Quackenbush, and G. C. Yuan, “Passing Messages between Biological Networks to Refine Predicted Interactions,” *PLoS One*, vol. 8, no. 5, May 2013.

[3] H. Zhao *et al.*, “LncTarD: A manually-curated database of experimentally-supported functional lncRNA-target regulations in human diseases,” *Nucleic Acids Res.*, vol. 48, no. D1, pp. D118–D126, 2020.

[4] D. Szklarczyk *et al.*, “The STRING database in 2017: Quality-controlled protein-protein association networks, made broadly accessible,” *Nucleic Acids Res.*, vol. 45, no. D1, pp. D362–D368, 2017.

[5] L. Cowen, T. Ideker, B. J. Raphael, and R. Sharan, “Network propagation: A universal amplifier of genetic associations,” *Nat. Rev. Genet.*, vol. 18, no. 9, pp. 551–562, 2017.

[6] M. D. M. Leiserson *et al.*, “Pan-cancer network analysis identifies combinations of rare somatic mutations across pathways and protein complexes,” *Nat. Genet.*, vol. 47, no. 2, pp. 106–114, Jan. 2015.

[7] Z. Liu and J. Luo, “Genome-wide predicting disease-related protein complexes by walking on the heterogeneous network based on data integration and laplacian normalization,” *Comput. Biol. Chem.*, vol. 69, pp. 41–47, 2017.

[8] G. Yu, L. G. Wang, Y. Han, and Q. Y. He, “ClusterProfiler: An R package for comparing biological themes among gene clusters,” *Omi. A J. Integr. Biol.*, vol. 16, no. 5, pp. 284–287, May 2012.

[9] A. Liberzon, A. Subramanian, R. Pinchback, H. Thorvaldsdóttir, P. Tamayo, and J. P. Mesirov, “Molecular signatures database (MSigDB) 3.0,” *Bioinformatics*, vol. 27, no. 12, pp. 1739–1740, 2011.

[10] D. N. Slenter *et al.*, “WikiPathways: A multifaceted pathway database bridging metabolomics to other omics research,” *Nucleic Acids Res.*, vol. 46, no. D1, pp. D661–D667, 2018.

[11] X. Robin *et al.*, “pROC: An open-source package for R and S+ to analyze and compare ROC curves,” *BMC Bioinformatics*, vol. 12, no. 1, p. 77, Mar. 2011.

[12] X. Guo *et al.*, “Long non-coding RNAs function annotation: A global prediction method based on bi-colored networks,” *Nucleic Acids Res.*, vol. 41, no. 2, Jan. 2013.

[13] K. Liu, Z. Yan, Y. Li, and Z. Sun, “Linc2GO: A human LincRNA function annotation resource based on ceRNA hypothesis,” *Bioinformatics*, vol. 29, no. 17, pp. 2221–2222, Sep. 2013.

[14] C. Park, N. Yu, I. Choi, W. Kim, and S. Lee, “LncRNAtor: A comprehensive resource for functional investigation of long non-coding RNAs,” *Bioinformatics*, vol. 30, no. 17, pp. 2480–2485, Sep. 2014.

[15] Q. Jiang *et al.*, “LncRNA2Function: A comprehensive resource for functional investigation of human lncRNAs based on RNA-seq data,” *BMC Genomics*, vol. 16, no. 3, Jan. 2015.

[16] J. Han *et al.*, “LncRNAs2Pathways: Identifying the pathways influenced by a set of lncRNAs of interest based on a global network propagation method,” *Sci. Rep.*, vol. 7, no. 1, pp. 1–14, Apr. 2017.
